# Supplementary material for: Nanoparticulate cell-free DNA scavenger for treating inflammatory bone loss in periodontitis
Source: Nat Commun. 2022 Oct 7;13:5925. doi: 10.1038/s41467-022-33492-6 (PMC9546917; doi:10.1038/s41467-022-33492-6)
Supplement: Supplementary file 1 — Supplementary Information [file 41467_2022_33492_MOESM1_ESM.pdf]

# Supplementary Materials

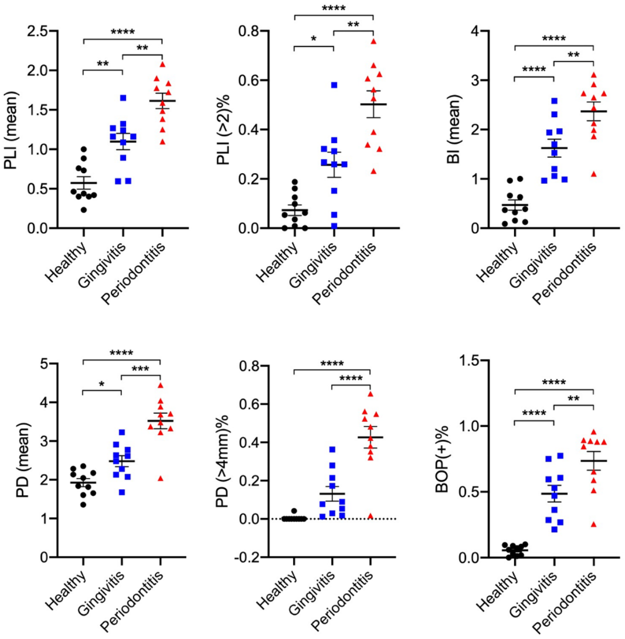


**Supplementary Fig. 1. Parameters of periodontal examination**

Parameters of periodontal examination of healthy volunteers (n=10), gingivitis patients (n=10) and periodontitis patients (n=10). Data are means ± SEM; differences were assessed by one-way analysis of variance and Tukey's multiple comparisons test. **P*<0.05, ***P*<0.01, ****P*<0.001, *****P*<0.0001. PLI (mean), *P*=0.0014 between Healthy and Gingivitis, *P*<0.0001 between Healthy and Periodontitis, *P*=0.0017 between Gingivitis and Periodontitis; PLI (>2)%, *P*=0.0193 between Healthy and Gingivitis, *P*<0.0001 between Healthy and Periodontitis, *P*=0.0018 between Gingivitis and Periodontitis; BI (mean), *P*<0.0001 between Healthy and Gingivitis, *P*<0.0001 between Healthy and Periodontitis, *P*=0.0088 between Gingivitis and Periodontitis; PD (mean), *P*=0.0446 between Healthy and Gingivitis, *P*<0.0001 between Healthy and Periodontitis, *P*=0.0002 between Gingivitis and Periodontitis; PD (>4mm)%, *P*<0.0001 between Healthy and Periodontitis, *P*<0.0001 between Gingivitis and Periodontitis; BOP(+)%, *P*<0.0001 between Healthy and Gingivitis, *P*<0.0001 between Healthy and Periodontitis, *P*=0.0098 between Gingivitis and Periodontitis. PLI: plaque index (1-3); BI: bleeding index (1-5); PD: probing depth (mm); BOP (+): bleeding on probing. "Mean" value is the arithmetic mean of the records of all examined sites. "(>n)%" is the percentage of site with parameter greater than "n" to the total number of sites.

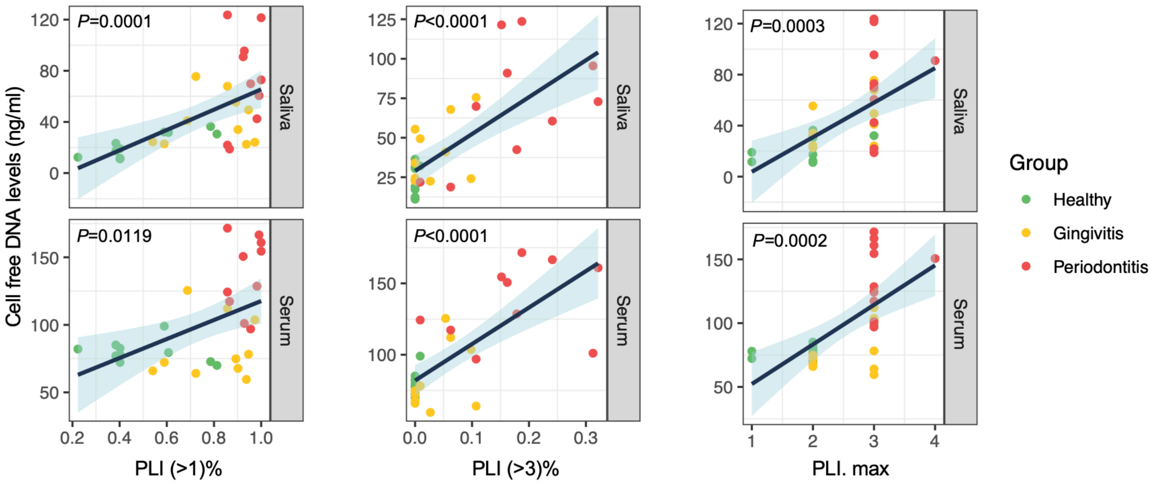


**Supplementary Fig. 2. Correlation of cfDNA levels with plaque index.**

Scatter plot of cfDNA levels and plaque index. The color of the dot represents the group of subjects. The dark blue line is the fitted regression line, and the light blue shading around it is the 95% confidence interval. PLI: plaque index. "(>n)%" is the percentage of site with parameter greater than "n" to the total number of sites. "Max" is the maximum of all sites.

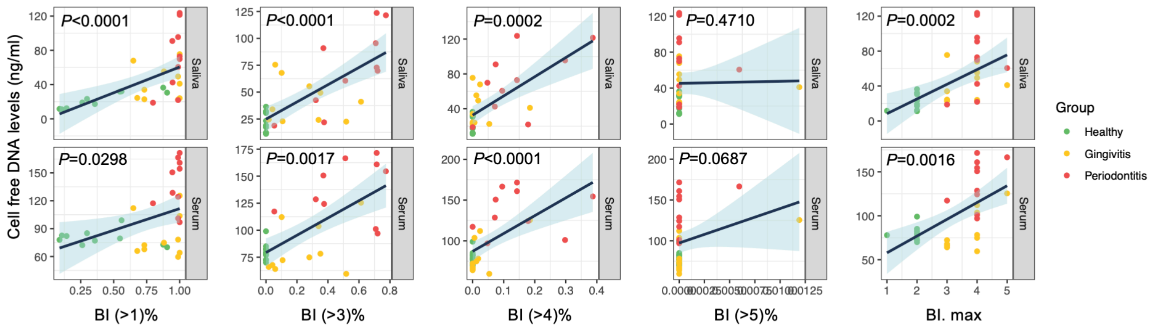


**Supplementary Fig. 3. Correlation of cfDNA levels with bleeding index.**

Scatter plot of cfDNA levels and bleeding index. The color of the dot represents the group of subjects. The dark blue line is the fitted regression line, and the light blue shading around it is the 95% confidence interval. BI: bleeding index. "(>n)%" is the percentage of site with parameter greater than "n" to the total number of sites. "Max" is the maximum of all sites.

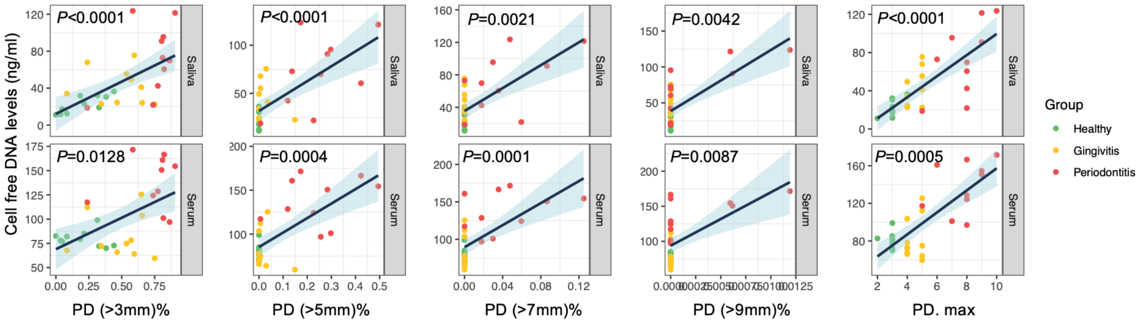


**Supplementary Fig. 4. Correlation of cfDNA levels with probing depth.**

Scatter plot of cfDNA levels and probing depth. The color of the dot represents the group of subjects. The dark blue line is the fitted regression line, and the light blue shading around it is the 95% confidence interval. PD: probing depth. "(>n)%" is the percentage of site with parameter greater than "n" to the total number of sites. "Max" is the maximum of all sites.

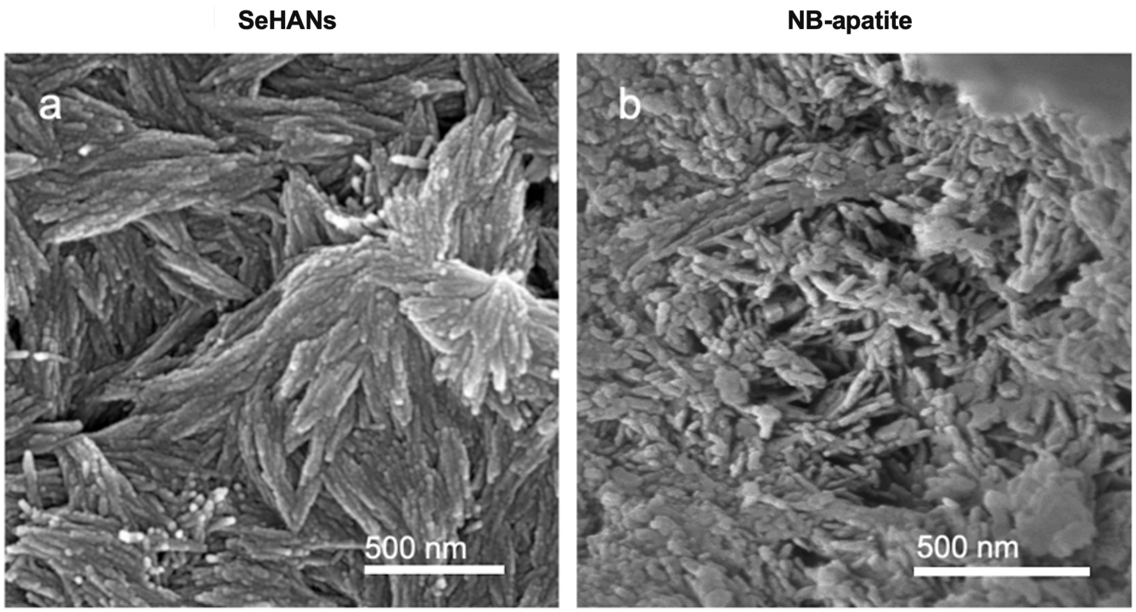


**Supplementary Fig. 5. Morphology comparison of synthetic SeHANs and natural bone (NB)-apatite by SEM.**

Representative images were from 3 independent experiments.

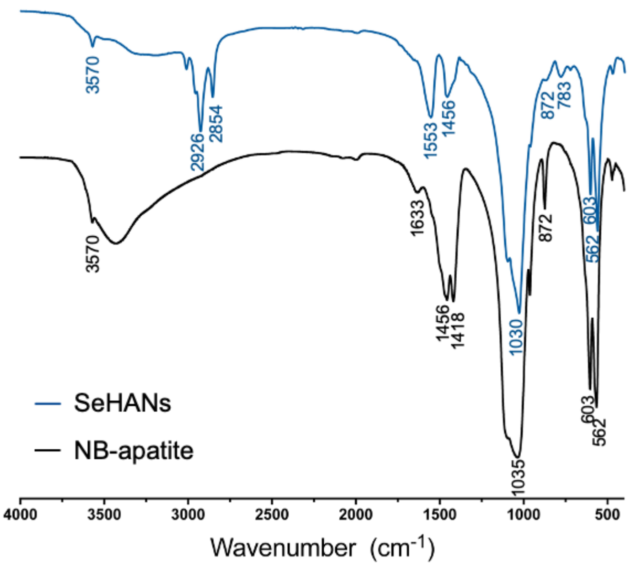


**Supplementary Fig. 6. FTIR spectra of SeHANs and natural bone (NB)-apatite.**
The typical bands of $PO_4^{3-}$ (1200-900, 603, 562 $cm^{-1}$) and $OH^-$ (3570 $cm^{-1}$) are present in both of SeHANs and NB-apatite. Carbonate bands at 872 $cm^{-1}$ and in the range of 1600-1400 $cm^{-1}$ are also found in both of synthetic SeHANs and NB-apatite.

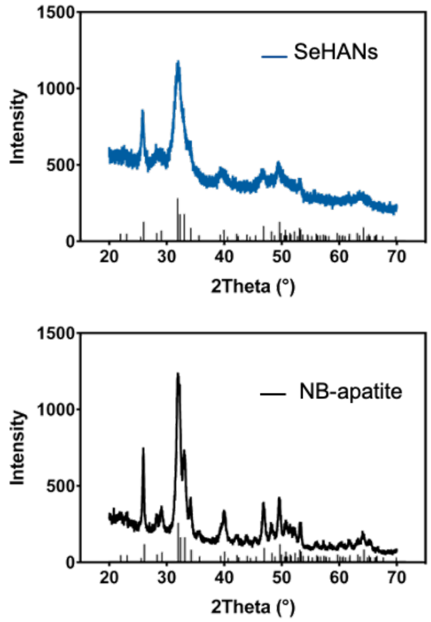


**Supplementary Fig. 7. XRD patterns of synthetic SeHANs and natural bone (NB)-apatite.**

(Top) XRD pattern of synthetic SeHANs. (Bottom) XRD pattern of NB-apatite.

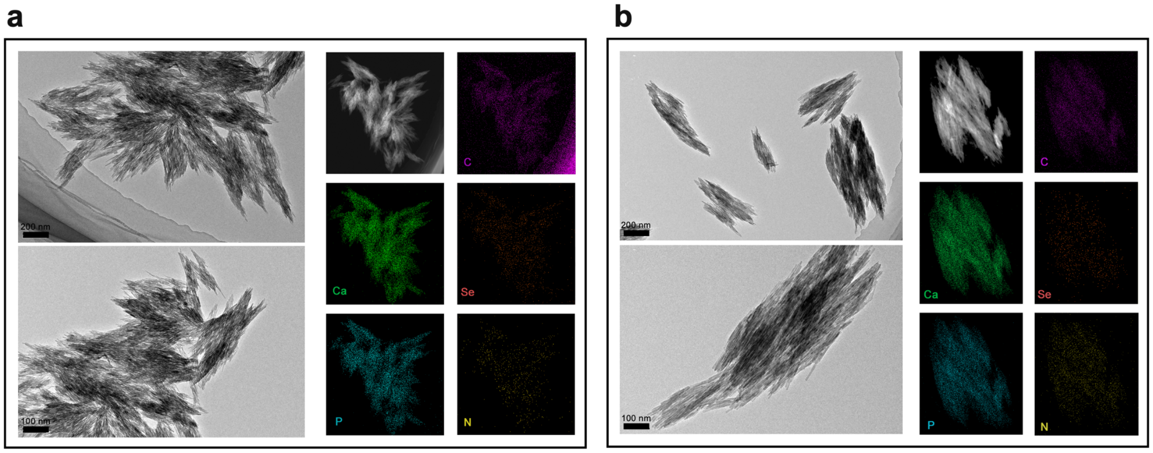


**Supplementary Fig. 8. TEM images and elemental mapping analysis of SeHANs and G3@SeHANs.**

TEM images and elemental mapping analyses of (a) SeHANs and (b) G3@SeHANs. Top, scale bars, 200 nm; Bottom, scale bars, 100 nm. Elemental maps of carbon (C, purple), calcium (Ca, green), Se (red), phosphorus (P, blue), and nitrogen (N, yellow) are shown. Representative images were from 3 independent experiments.

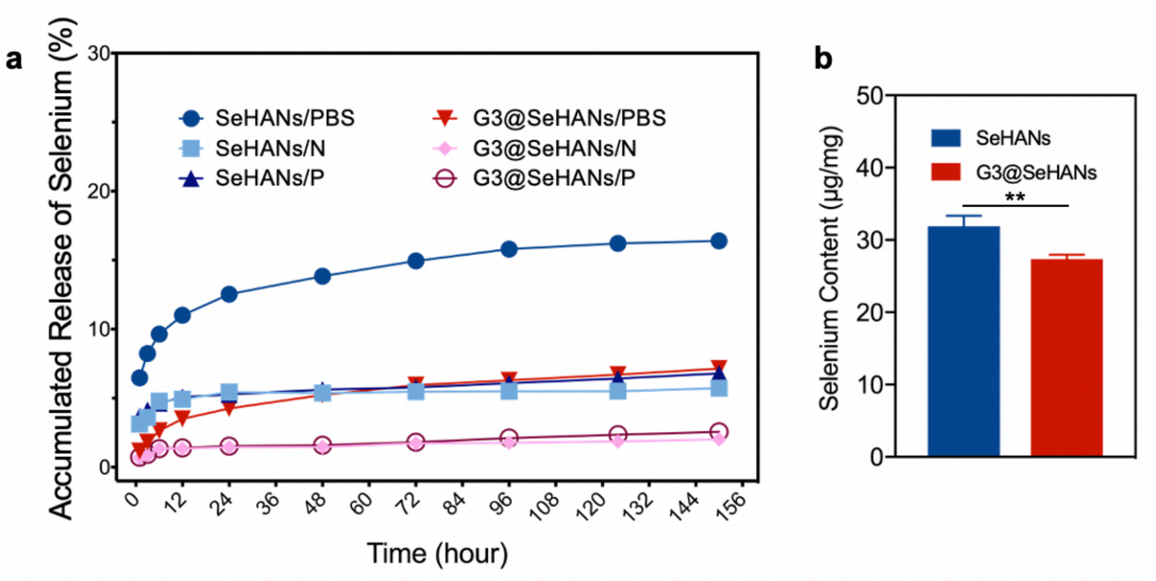


**Supplementary Fig. 9. Biodegradation and selenium release rate of SeHANs and G3@SeHANs.**

The amount of selenium in synthesized nanoparticles was quantified using ICP-OES. (a) Selenium release by SeHANs and G3@SeHANs is indicative of their biodegradation and was detected by ICP-OES in three different solutions: PBS pH 7.4, saliva from healthy people (N), and saliva from periodontitis patients (P). (b) Selenium content of SeHANs and G3@SeHANs. Data are means ± SEM; differences were assessed by Two-tailed Student's *t*-test (n=3) ($P$=0.0075).

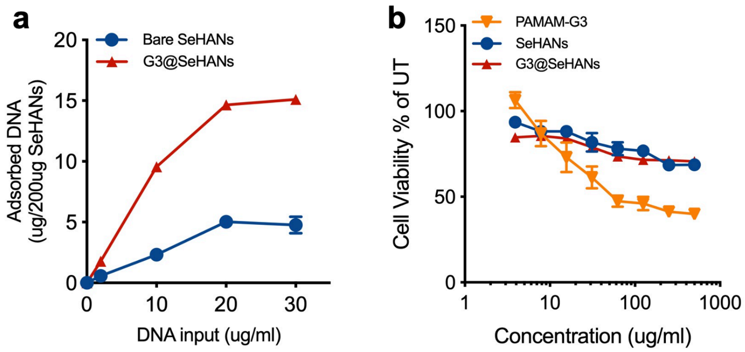


**Supplementary Fig. 10. DNA binding efficiency and cytotoxicity of SeHANs and G3@SeHANs.**

(a) DNA binding efficiency of SeHANs and G3@SeHANs. (b) Viability of RAW 264.7 cells treated with PAMAM-G3, SeHANs, or G3@SeHANs at various concentrations for 24 h. Data are means ± SEM; n=3.

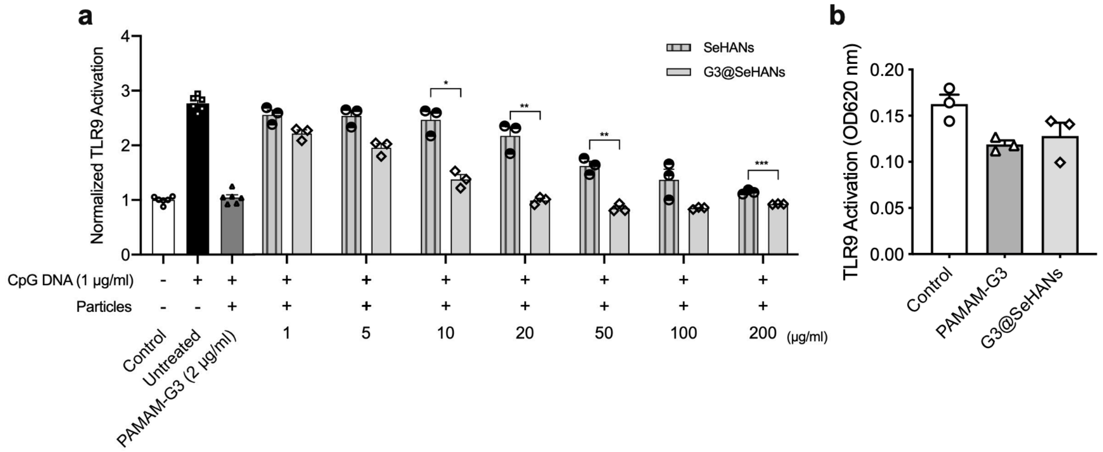


**Supplementary Fig. 11. Effects of bare SeHANs, G3@SeHANs, and PAMAM-G3 on cfDNA-induced proinflammatory response.**

Activation of HEK-TLR9 reporter cells by (a) CpG DNA (1 μg/mL) in the absence or presence of SeHANs and G3@SeHANs at 1, 5, 10, 20, 50, 100, and 200 μg/mL for 24 h, and by (b) bare PAMAM-G3 (2 μg/mL) and G3@SeHANs (10 μg/mL) for 24 h. All data are means ± SEM; differences were assessed by one-way ANOVA with Tukey's multiple comparison test ($n$=3 independent experiments; *$P$<0.05, **$P$<0.01, ***$P$<0.001, ****$P$<0.0001) (a: 10, $P$=0.0032; 20, $P$=0.0021; 50, $P$=0.0013; 200, $P$=0.0007).

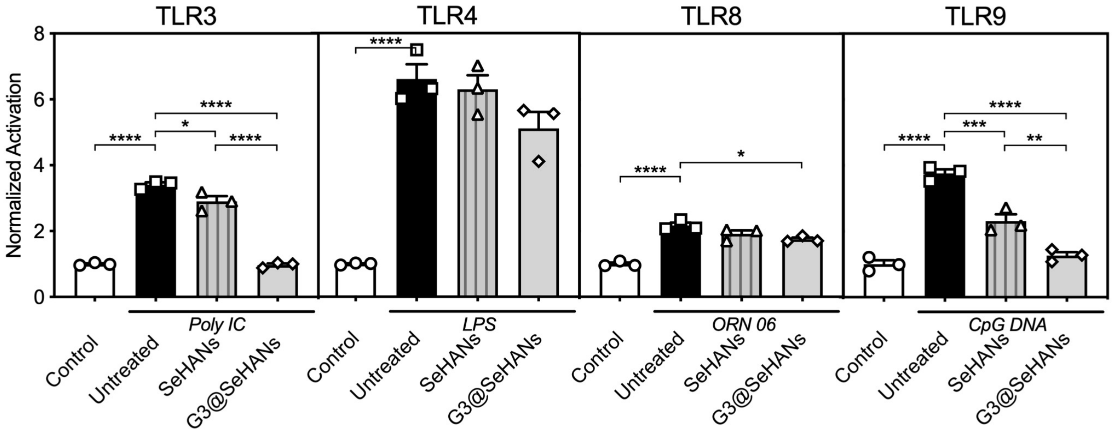


**Supplementary Fig. 12. G3@SeHANs limit the proinflammatory response *in vitro* through different TLR receptors.**

Activation of HEK-TLR3, -TLR4, -TLR8, and -TLR9 reporter cells by poly(I:C) (1 μg/mL), LPS (1 ng/mL), ORN06 (500 ng/mL), and CpG DNA (1 μg/mL) in the absence or presence of PAMAM-G3 (2 μg/mL) or G3@SeHANs (10 μg/mL) for 24 h. Data are means ± SEM; differences were assessed by one-way ANOVA with Tukey's multiple comparison test; $n$=3 independent experiments; *$P$<0.05, **$P$<0.01, ***$P$<0.001, ****$P$<0.0001 (Poly IC, $P$<0.0001 between Control and Untreated, $P$=0.0199 between Untreated and SeHANs, $P$<0.0001 between Untreated and G3@SeHANs, $P$<0.0001 between SeHANs and G3@SeHANs; LPS, $P$<0.0001 between Control and Untreated; ORN 06, $P$<0.0001 between Control and Untreated, $P$=0.0217 between Untreated and G3@SeHANs; CpG DNA, $P$<0.0001 between Control and Untreated, $P$=0.0005 between Untreated and SeHANs, $P$<0.0001 between Untreated and G3@SeHANs, $P$=0.0039 between SeHANs and G3@SeHANs).

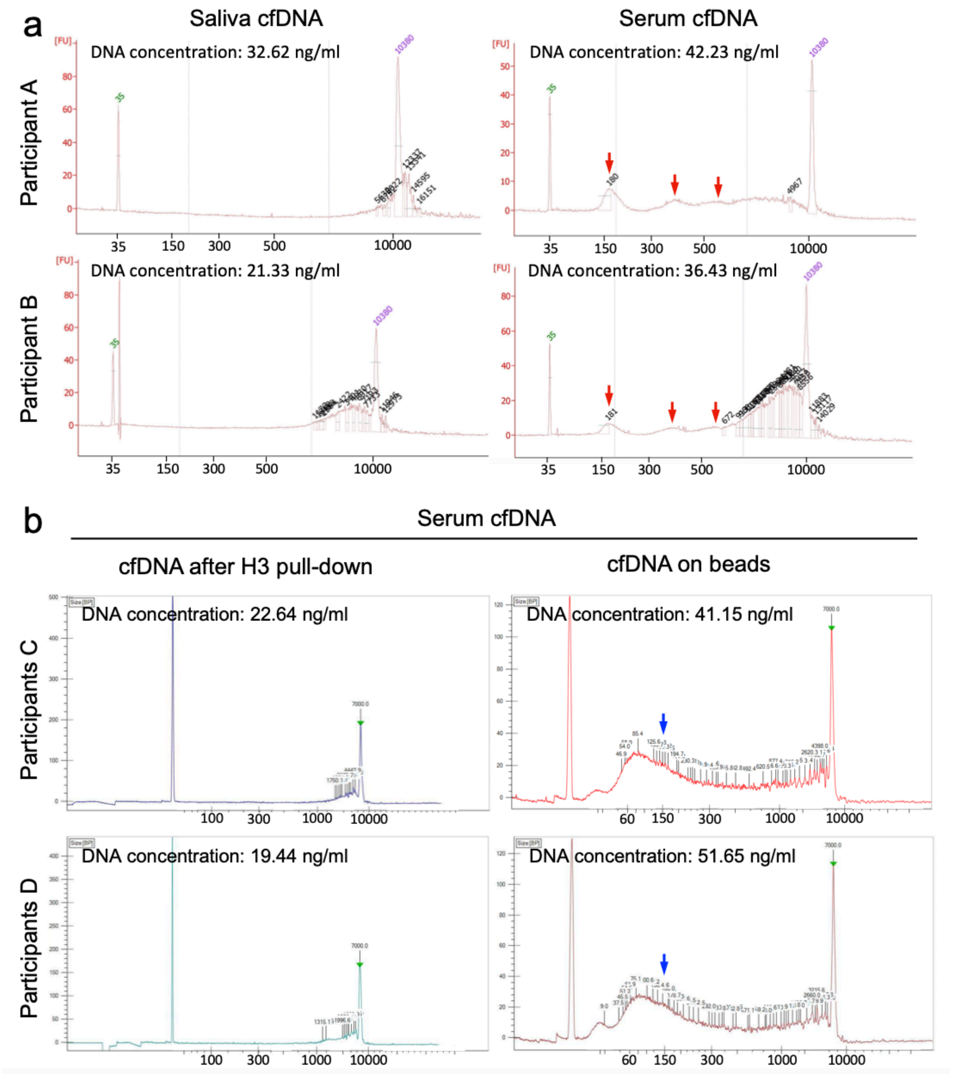


**Supplementary Fig. 13. The size distribution of cfDNA fragments in saliva and serum and serum cfDNA detected after histone H3 pull-down**

(a) The cfDNA was extracted from the serum and saliva of two healthy participants. The same DNA samples were used for DNA concentration determination and detection of DNA fragment size distribution, respectively. Serum cfDNA samples were diluted twice before analysis. The red arrows indicate the characteristic peaks in the size distribution of serum cfDNA. (b) Serum samples from another two healthy participants were collected for histone H3 pull-down. The left column shows the size distribution of cfDNA extracted from the remaining serum sample after histone H3 pull-down; the right column shows the size distribution of DNA extracted from the magnetic beads (captured by histone H3 antibody). The blue arrows indicate the small peaks around 150bp.

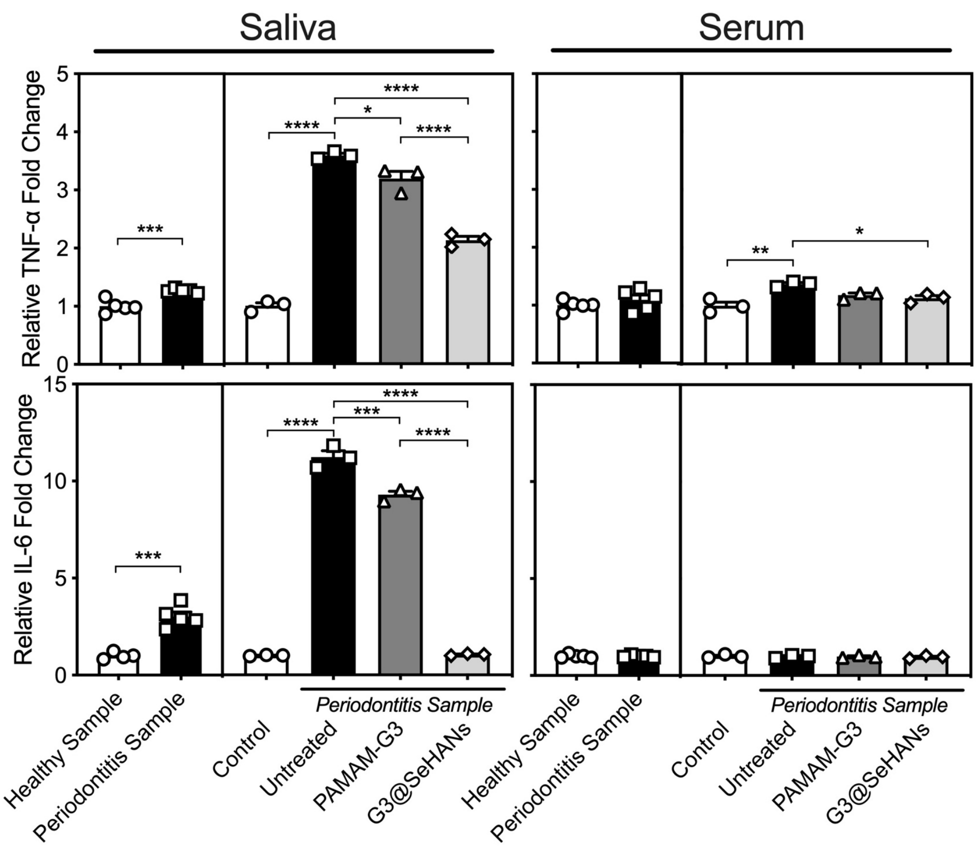


**Supplementary Fig. 14. G3@SeHANs block the proinflammatory response of saliva and serum in RAW 264.7 macrophages.**

TNF-α and IL-6 expression by RAW 264.7 macrophages induced by healthy human saliva, periodontitis patient saliva, healthy human serum, or periodontitis patient serum in the absence or presence of PAMAM-G3 (2 μg/mL) or G3@SeHANs (10 μg/mL) for 24 h. Data are means ± SEM; **P*<0.05, ***P*<0.01, ****P*<0.001, *****P*<0.0001 by two-tailed Student's *t*-test (*n*=5 independent experiments) and one-way ANOVA with Tukey's multiple comparison test (*n*=3 independent experiments) (Saliva TNF-α, *P*=0.0007 between Healthy Sample and Periodontitis Sample, *P*<0.0001 between Control and Untreated, *P*=0.0262 between Untreated and PAMAM-G3, *P*<0.0001 between Untreated and G3@SeHANs, *P*<0.0001 between PAMAM-G3 and G3@SeHANs; Serum TNF-α, *P*=0.0018 between Control and Untreated, *P*=0.0239 between Untreated and G3@SeHANs; Saliva IL-6, *P*=0.0002 between Healthy Sample and Periodontitis Sample, *P*<0.0001 between Control and Untreated, *P*=0.0004 between Untreated and PAMAM-G3, *P*<0.0001 between Untreated and G3@SeHANs, *P*<0.0001 between PAMAM-G3 and G3@SeHANs).

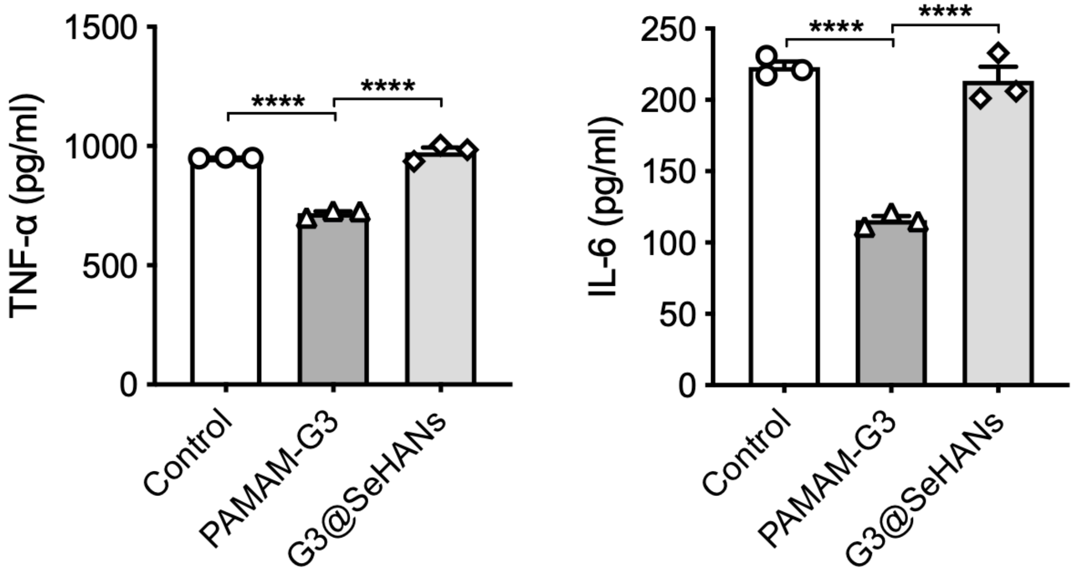


**Supplementary Fig. 15. Effects of bare SeHANs and G3@SeHANs on RAW 264.7 macrophages.**

TNF-α and IL-6 expression by RAW 264.7 macrophages treated witt PAMAM-G3 (2 μg/mL), or G3@SeHANs (10 μg/mL) without agonist for 24 h. No item was added to the control group. All data are means ± SEM; differences were assessed by one-way ANOVA with Tukey's multiple comparison test ($n$=3 independent experiments; *$P$<0.05, **$P$<0.01, ***$P$<0.001, ****$P$<0.0001) (TNF-α, $P$<0.0001 between Control and PAMAM-G3, $P$<0.0001 between PAMAM-G3 and G3@SeHANs; IL-6, $P$<0.0001 between Control and PAMAM-G3, $P$<0.0001 between PAMAM-G3 and G3@SeHANs).

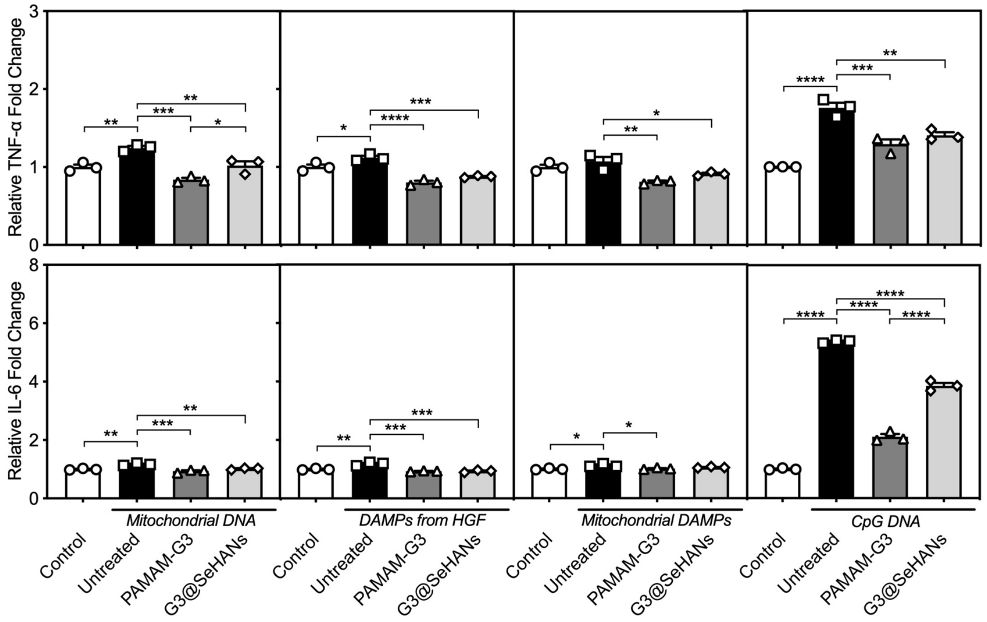


**Supplementary Fig. 16. G3@SeHANs block the proinflammatory response of DAMPs and DNA in RAW 264.7 macrophages.**

RAW 264.7 macrophages were stimulated with mtDNA, DAMPs from gingival fibroblasts, DAMPs from mitochondria, or CpG DNA in the absence or presence of PAMAM-G3 (2 μg/mL) or G3@SeHANs (10 μg/mL) for 24 h. Supernatants were assayed for TNF-α and IL-6 by ELISA. Data are means ± SEM; differences were assessed by one-way ANOVA with Tukey's multiple comparison test; $n$=3 independent experiments; *$P$<0.05, **$P$<0.01, ***$P$<0.001, ****$P$<0.0001 (TNF-α-Mitochondrial DNA, $P$=0.0057 between Control and Untreated, $P$=0.0002 between Untreated and PAMAM-G3, $P$=0.0093 between Untreated and G3@SeHANs, $P$=0.0317 between PAMAM-G3 and G3@SeHANs; TNF-α-DAMPs from HGF, $P$=0.0296 between Control and Untreated, $P$<0.0001 between Untreated and PAMAM-G3, $P$=0.0004 between Untreated and G3@SeHANs; TNF-α-Mitochondrial DAMPs, $P$=0.0030 between Untreated and PAMAM-G3, $P$=0.0438 between Untreated and G3@SeHANs; TNF-α-CpG DNA, $P$<0.0001 between Control and Untreated, $P$=0.0006 between Untreated and PAMAM-G3, $P$=0.0038 between Untreated and G3@SeHANs; IL-6-Mitochondrial DNA, $P$=0.0041 between Control and Untreated, $P$=0.0005 between Untreated and PAMAM-G3, $P$=0.0070 between Untreated and G3@SeHANs; IL-6-DAMPs from HGF, $P$=0.0021 between Control and Untreated, $P$=0.0003 between Untreated and PAMAM-G3, $P$=0.0003 between Untreated and G3@SeHANs; IL-6-Mitochondrial DAMPs, $P$=0.0141 between Control and Untreated, $P$=0.0280 between Untreated and PAMAM-G3; IL-6-CpG DNA, $P$<0.0001 between Control and Untreated, $P$<0.0001 between Untreated and PAMAM-G3, $P$<0.0001 between Untreated and G3@SeHANs, $P$<0.0001 between PAMAM-G3 and G3@SeHANs).

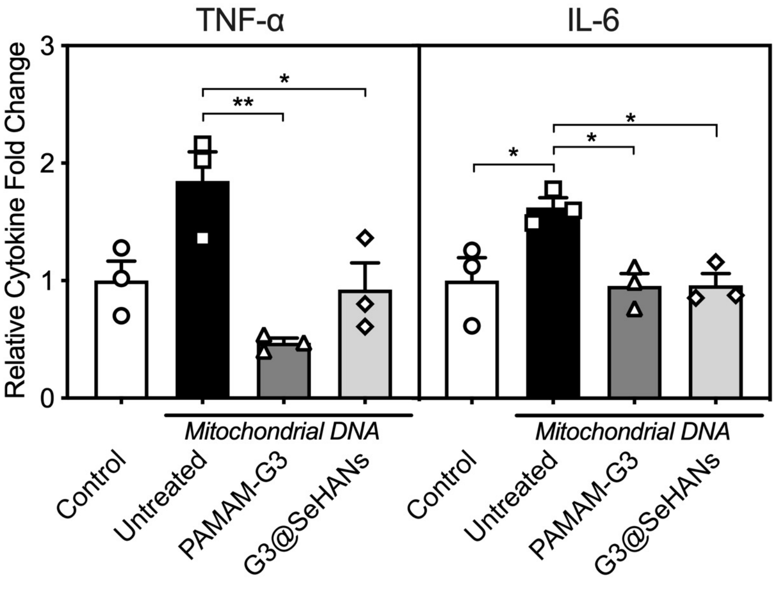


**Supplementary Fig. 17. G3@SeHANs block the mtDNA-driven proinflammatory response of THP-1 macrophages.**

THP-1 macrophages were stimulated with mtDNA in the absence or presence of PAMAM-G3 (2 μg/mL) or G3@SeHANs (10 μg/mL) for 24 h. Supernatants were assayed for TNF-α and IL-6 by ELISA. Data are means ± SEM; differences were assessed by one-way ANOVA with Tukey's multiple comparison test; $n$=3 independent experiments; *$P$<0.05 (TNF-α, $P$=0.0037 between Untreated and PAMAM-G3, $P$=0.0342 between Untreated and G3@SeHANs; IL-6, $P$=0.0362 between Control and Untreated, $P$=0.0260 between Untreated and PAMAM-G3, $P$=0.0272 between Untreated and G3@SeHANs).

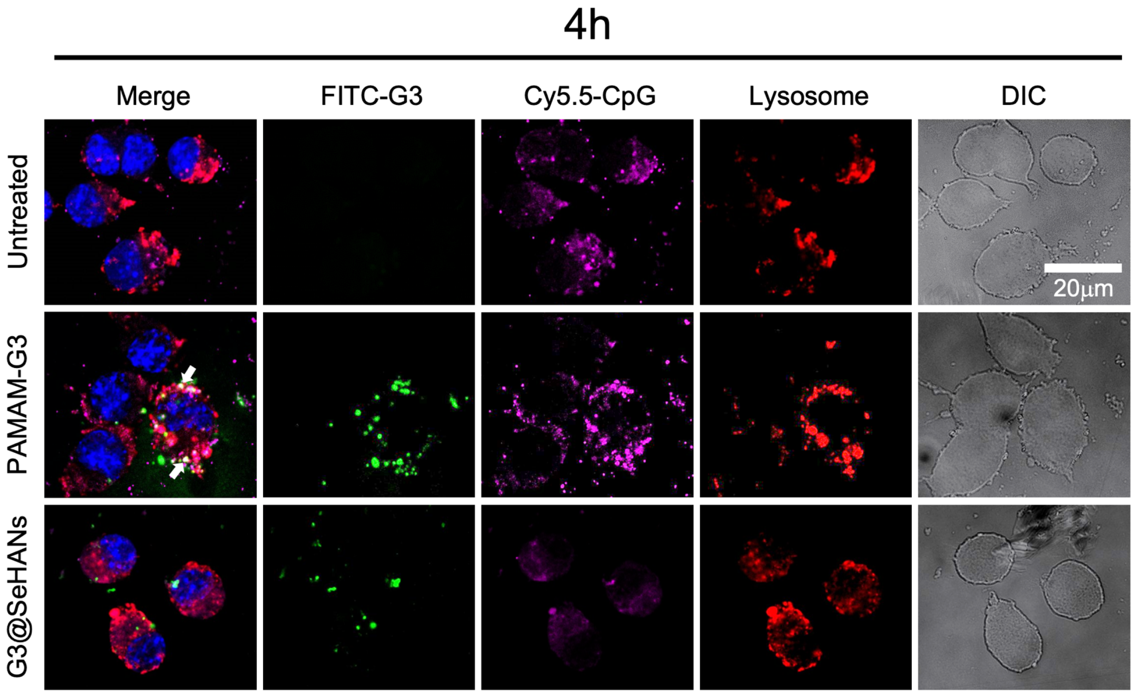


**Supplementary Fig. 18. Intracellular localization of intracellular CpG and cationic materials after a 4 h incubation.**

Enlarged images showing the intracellular localization of intracellular CpG and cationic materials in RAW 264.7 cells after a 4 h incubation. Scale bar, 20 μm. The colocalization of CpG and cationic materials appeared as white spots, indicated by the arrows. Representative images were from 3 independent experiments.

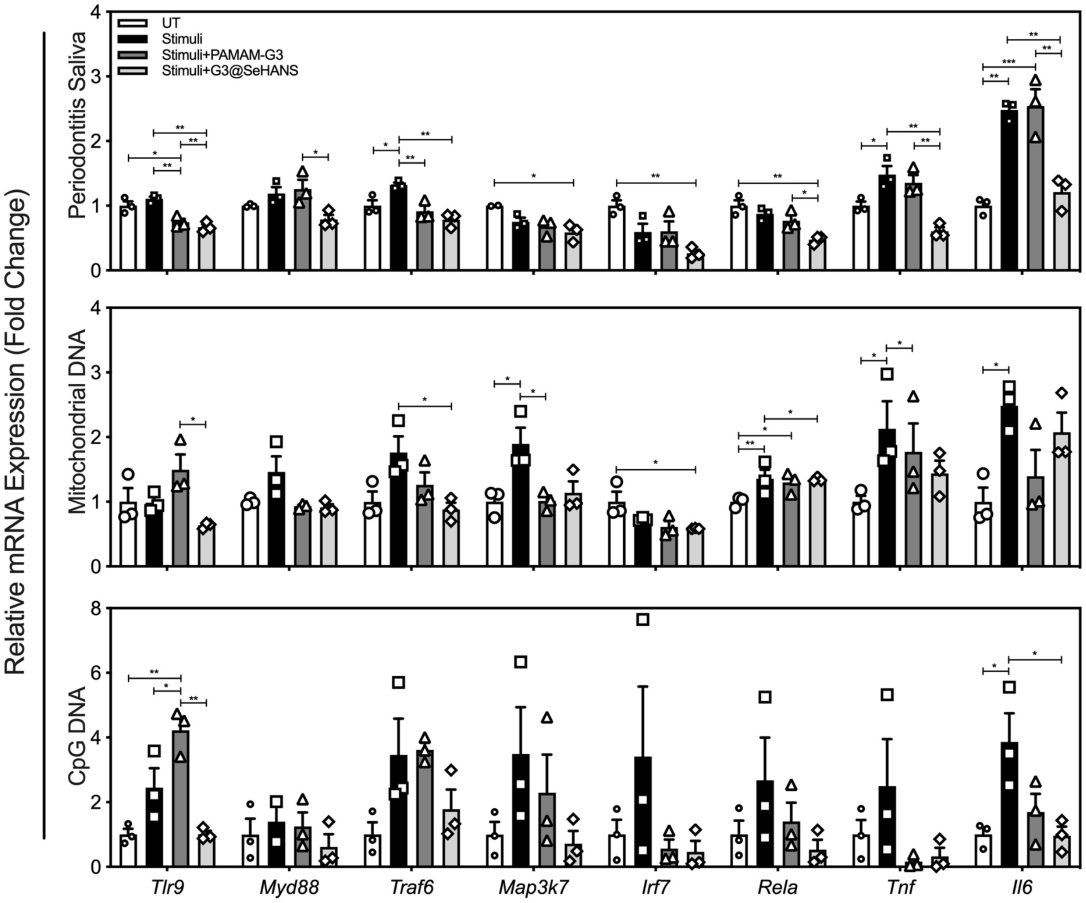


**Supplementary Fig. 19. Relative gene expression of the TLR9-NFκB pathway in RAW 264.7 cells with medium containing pathological DNA and cfDNA scavengers.**

Relative gene expression of the TLR9-NFκB pathway (*Tlr9*, *Myd88*, *Traf6*, *Map3k7*, *Irf7*, *Rela*, *Tnf*, and *Il6*) in RAW 264.7 cells after a 24 h incubation with medium containing pathological DNA and cfDNA scavengers. The stimulus were added after 30 min of incubation with scavengers. Data are means ± SEM; $n$=3 samples per group; *$P$<0.05, **$P$<0.01, ***$P$<0.001 by one-way ANOVA with Tukey's multiple comparison test. UT, untreated. Periodontitis Saliva: *Tlr9,* $P$=0.0249 between UT and Stimuli+PAMAM-G3, $P$=0.0074 between UT and Stimuli+G3@SeHANs, $P$=0.0057 between Stimuli and Stimuli+PAMAM-G3, $P$=0.0019 between Stimuli and Stimuli+G3@SeHANs; *Myd88*, $P$=0.0465 between Stimuli+PAMAM-G3 and Stimuli+G3@SeHANs; *Traf6*, $P$=0.0462 between UT and Stimuli, $P$=0.0174 between Stimuli and Stimuli+PAMAM-G3, $P$=0.0038 between Stimuli and Stimuli+G3@SeHANs; *Map3k7*, $P$=0.0349 between UT and Stimuli+G3@SeHANs; *Irf7*, $P$=0.0094 between UT and Stimuli+G3@SeHANs; *Rela*, $P$=0.0038 between UT and Stimuli+G3@SeHANs, $P$=0.0163 between Stimuli and Stimuli+G3@SeHANs; *Tnf*, $P$=0.0367 between UT and Stimuli, $P$=0.0016 between Stimuli and Stimuli+G3@SeHANs, $P$=0.0037 between Stimuli+PAMAM-G3 and

Stimuli+G3@SeHANs; *Il6*, *P*=0.0009 between UT and Stimuli, *P*=0.0008 between UT and Stimuli+PAMAM-G3, *P*=0.0015 between Stimuli and Stimuli+G3@SeHANs, *P*=0.0014 between Stimuli+PAMAM-G3 and Stimuli+G3@SeHANs. Mitochondrial DNA: *Tlr9, P*=0.0359 between Stimuli+PAMAM-G3 and Stimuli+G3@SeHANs; *Traf6*, *P*=0.0448 between Stimuli and Stimuli+G3@SeHANs; *Map3k7*, *P*=0.0363 between UT and Stimuli, *P*=0.0363 between Stimuli and Stimuli+G3@SeHANs; *Irf7*, *P*=0.0442 between UT and Stimuli+G3@SeHANs; *Tnf*, *P*=0.0211 between UT and Stimuli, *P*=0.0457 between Stimuli and Stimuli+PAMAM-G3; *Il6*, *P*=0.0441 between UT and Stimuli. CpG DNA: *Tlr9, P*=0.0019 between UT and Stimuli+PAMAM-G3, *P*=0.0417 between Stimuli and Stimuli+PAMAM-G3, *P*=0.00019 between Stimuli+PAMAM-G3 and Stimuli+G3@SeHANs; *Il6*, *P*=0.0371 between UT and Stimuli, *P*=0.0371 between Stimuli and Stimuli+G3@SeHANs.

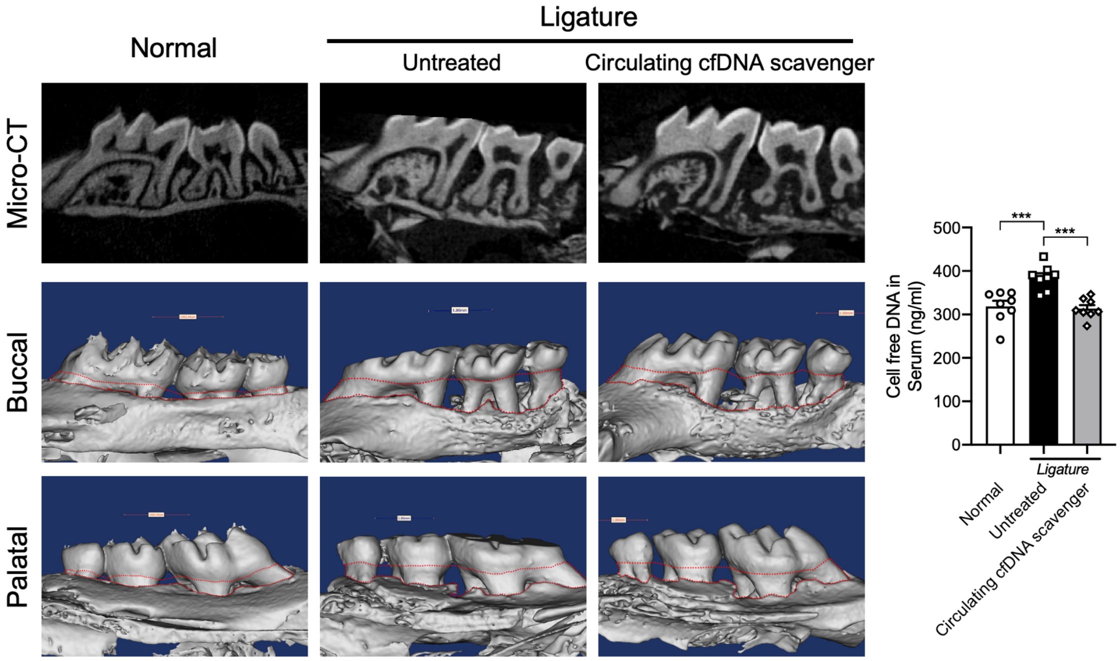


**Supplementary Fig. 20. Pilot animal study using circulating cfDNA-scavenging nucleic acid-binding nanoparticles.**

Circulating cfDNA-scavenging nucleic acid-binding nanoparticles (MSN-PEI) were applied in the ligature-induced periodontitis model. Circulating cfDNA level decreased, but there was no significant reduction of the alveolar bone loss. Data are means ± SEM; $n$=8 samples per group, *$P$<0.05, **$P$<0.01 by one-way ANOVA with Tukey's multiple comparison test ($P$=0.0007 between Normal and Untreated, $P$=0.0003 between Untreated and Circulating cfDNA scavenger).

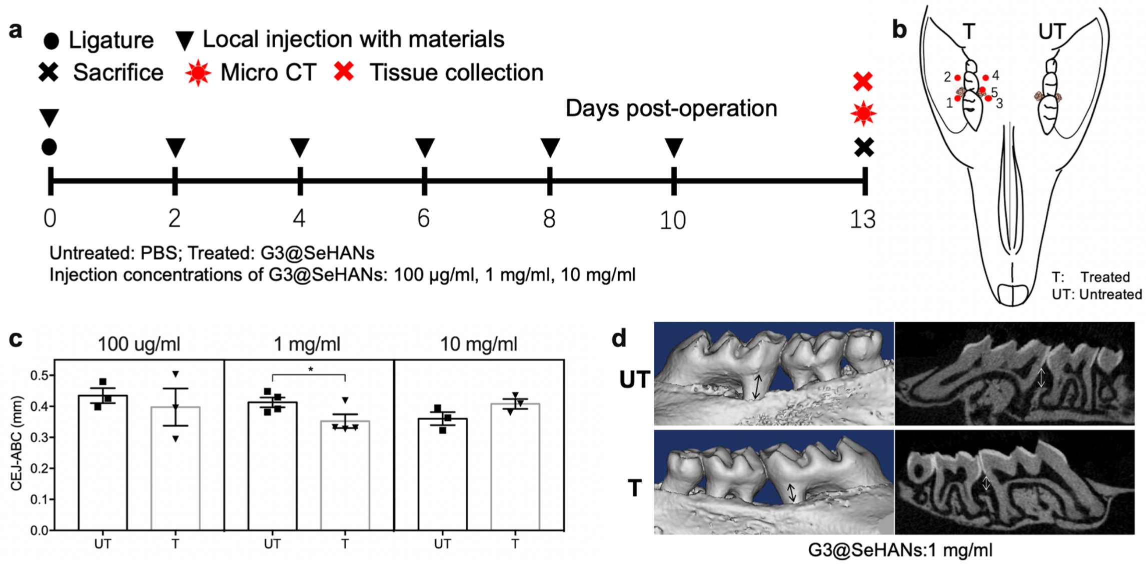


**Supplementary Fig. 21. Pilot animal study to determine G3@SeHANs dose.**

(a-b) Experimental schedule of the mouse study. The local administration of G3@SeHANs or PBS was performed by microinjection using a microsyringe into gingival tissue at five sites (5 μL/site) around the ligature every two days (on days 0, 2, 4, 6, 8, and 10) until the mice were sacrificed on day 13. For the pilot animal study, one side of the ligatures was treated with G3@SeHANs at three concentrations (100 μg/mL, 1 mg/mL, and 10 mg/mL) (treated, T), and the other side was injected with PBS (untreated, UT). (c) CEJ-ABC was used to measure bone loss. Different concentrations of G3@SeHANs were administered to determine the appropriate dose. Differences were assessed by two-tailed Student's *t*-test. Data are means ± SEM; $n$=3 samples per group for 100 μg/ml and 10 mg/ml, $n$=4 samples per group for 1 mg/ml; *$P$<0.05 (1 mg/ml, $P$=0.0226). (d) 3D reconstruction and CT scans of the bone loss in the T and UT groups.

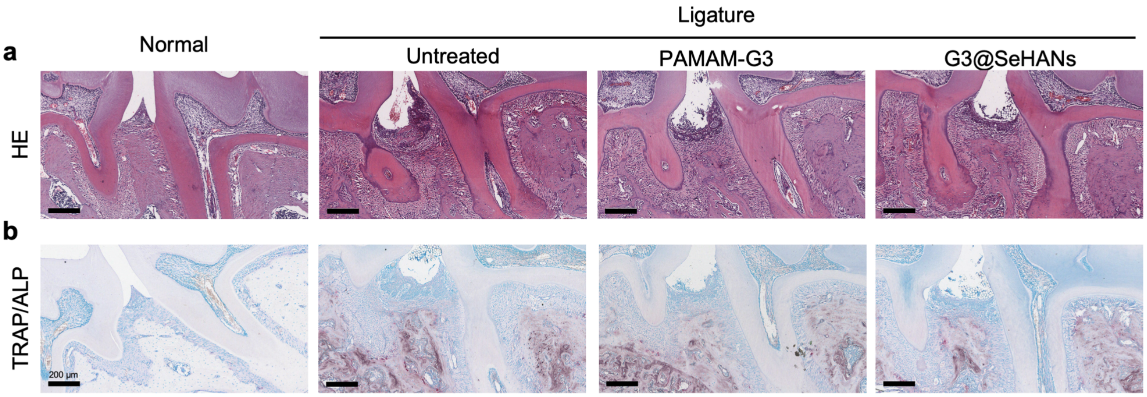


**Supplementary Fig. 22. H&E staining and TRAP/ALP staining of periodontal tissues at Day 15.**

(a) H&E staining of periodontal tissues on day 15 after G3@SeHANs administration (scale bars, 200 μm). Inflammatory cell infiltration in the epithelium and bone destruction were clearly evident in the untreated model, while treatment with G3@SeHANs efficiently prevented these pathological changes. (b) TRAP/ALP staining of periodontal tissues on day 15 after G3@SeHANs administration (scale bars, 200 μm). Images were representative of three independent mice.

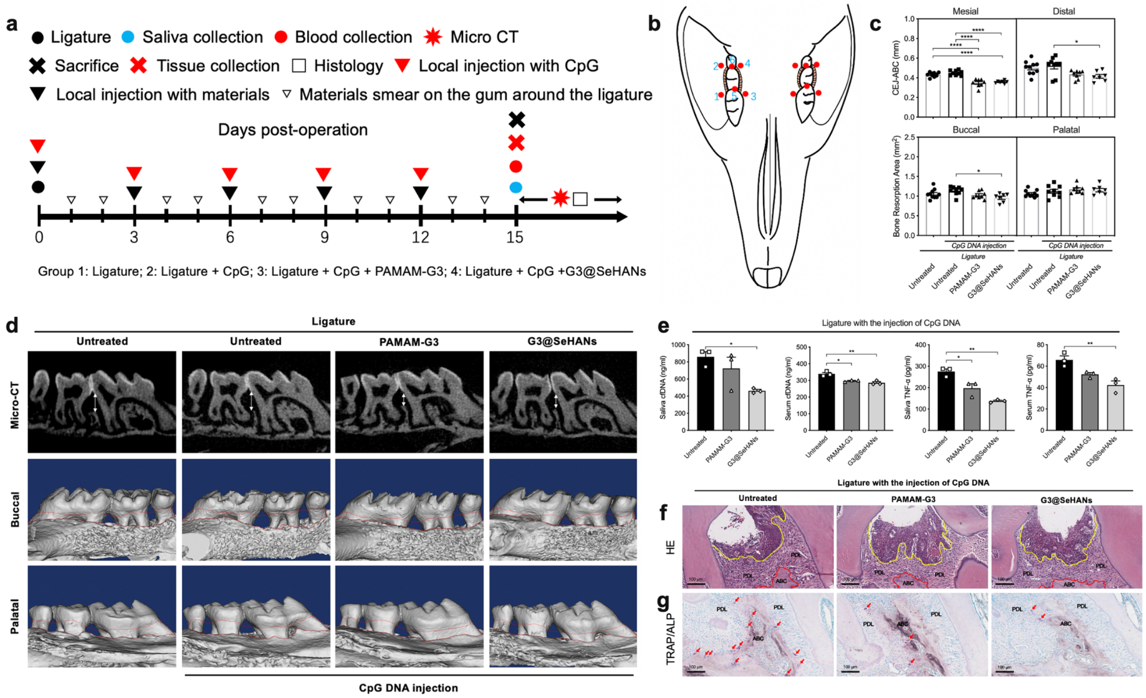

**Supplementary Fig. 23. cfDNA-enhanced inflammatory bone loss was alleviated by G3@SeHANs.**

(a-b) Experimental schedule of the mouse study. The local injection of CpG was performed in addition to ligature placement. The injection of CpG was performed as described above 30 min after the injection of materials or PBS. (c) The CEJ-ABC was used to measure bone loss. The mesial and distal CEJ-ABC (μm) of the maxillary second molar was recorded. The bone resorption area was defined as the area enclosed by the continuous line of the CEJ of the three molars and the ABC. Data are means ± SEM; **P*<0.05, *****P*<0.0001 by one-way ANOVA with Tukey's multiple comparison test (Mesial, *P*<0.0001 between Untreated and PAMAM-G3, *P*<0.0001 between Untreated and G3@SeHANs, *P*<0.0001 between Untreated-CpG and PAMAM-G3, *P*<0.0001 between Untreated-CpG and G3@SeHANs; Distal, *P*=0.0209 between Untreated-CpG and G3@SeHANs; Buccal, *P*=0.0140 between Untreated-CpG and G3@SeHANs). (d) 3D reconstruction and CT scans of the bone loss in different groups. (e) Saliva cfDNA level, serum cfDNA level, saliva TNF-α level, and serum TNF-α level 15 days postoperation. Data are means ± SEM; *n*=3 samples per group, **P*<0.05, ***P*<0.01 by one-way ANOVA with Tukey's multiple comparison test (Saliva cfDNA, *P*=0.0343 between Untreated and G3@SeHANs; Serum cfDNA, *P*=0.0105 between Untreated and PAMAM-G3, *P*=0.0032 between Untreated and G3@SeHANs; Saliva TNF-α, *P*=0.0193 between Untreated and PAMAM-G3, *P*=0.0011 between Untreated and G3@SeHANs; Serum TNF-α, *P*=0.0052 between Untreated and G3@SeHANs). (f) H&E staining of periodontal tissues on day 15 (scale bars, 100 μm). (g) TRAP/ALP staining of periodontal tissues on day 15 (scale bars, 100 μm). The number of

osteoclasts (red arrows) in the untreated group was higher than that in the scavenger-treated groups. Images (f, g) were representative of three independent mice.

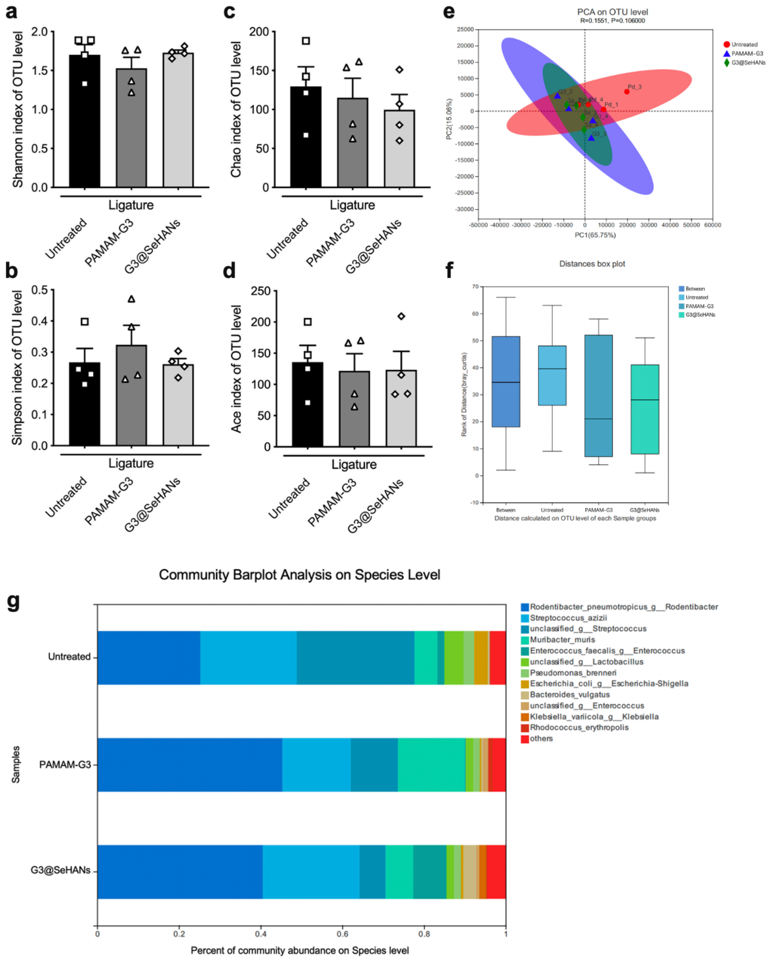


**Supplementary Fig. 24. Effects of G3@SeHANs on the oral microbiota of periodontitis mouse orally derived multispecies biofilms.**

(a-d) Shannon index, Chao index, Simpson index, and Ace index at the OTU level showing the diversity of bacterial communities in the oral cavity of ligature-induced periodontitis mice was unaffected by administration of G3@SeHANs. Data are means ± SEM; differences were assessed by one-way ANOVA with Tukey's multiple comparison test (*n*=4 samples per group). (e) In a principal component analysis (PCA) plot, samples from the untreated, PAMAM-G3, and G3@SeHAN groups clustered together, suggesting similar microbial community structures (*n*=4 samples per group). (f) Rank of the Bray-Curtis distance between or within groups showing between-group differences were similar to within-group differences (n=4). Statistical analyses were performed using ANOSIM. In box plot, center lines indicate median values. The lower and upper bounds represent 25th and 75th percentiles, respectively. The lower/upper whiskers represent minima/maxima no further than 1.5 times the interquartile range from the hinge. (g) Relative abundance of species-level taxa in the untreated, PAMAM-G3, and G3@SeHANs groups indicating the microbial compositions in the three groups were similar.

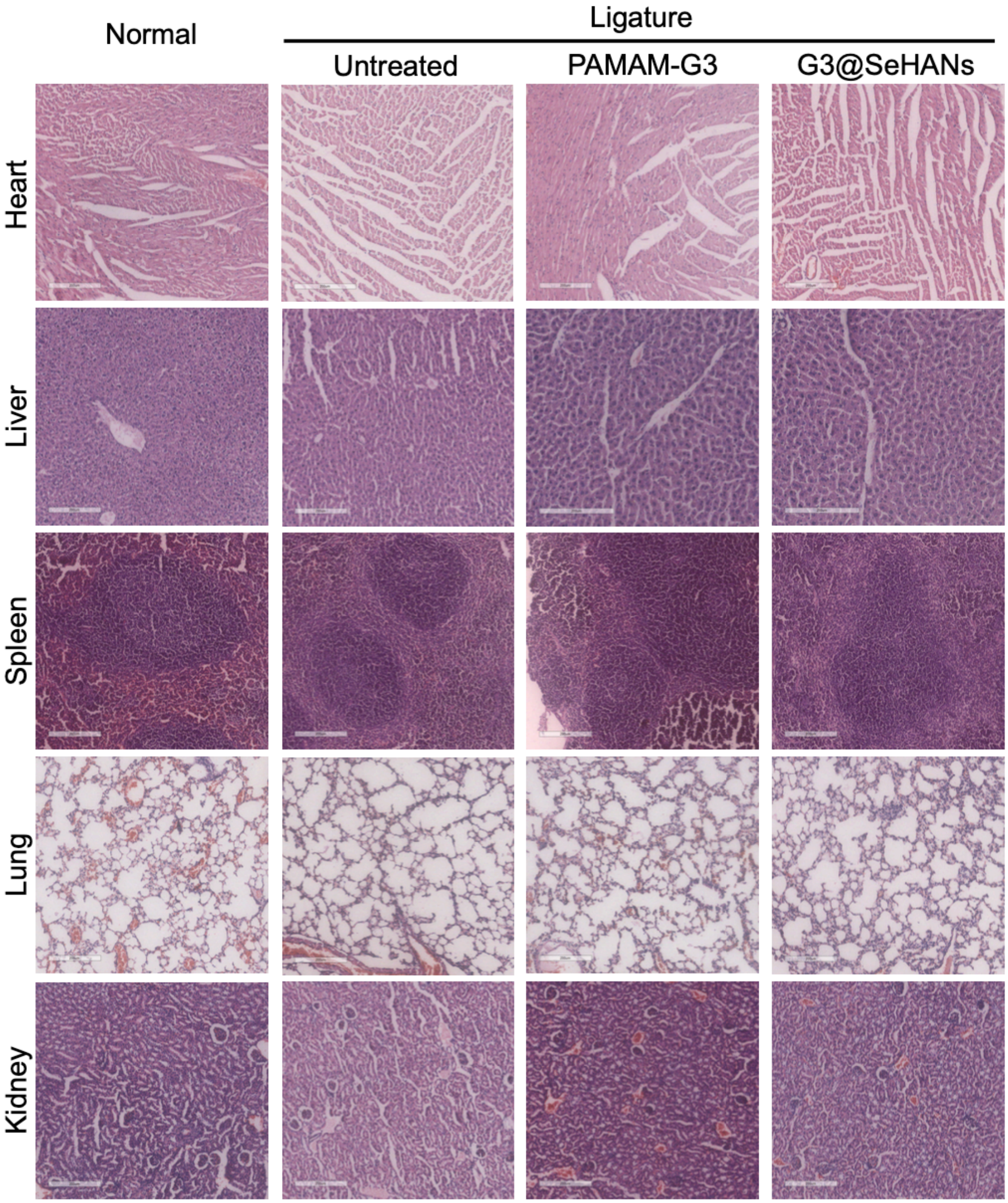


**Supplementary Fig. 25. H&E staining of multiple organs.**

Fifteen days after the ligature was placed, the heart, liver, spleen, lung and kidney were collected, stained with H&E and analyzed. Scale bars, 200 μm. No obvious pathological change was found in the four groups. Images were representative of three independent mice.

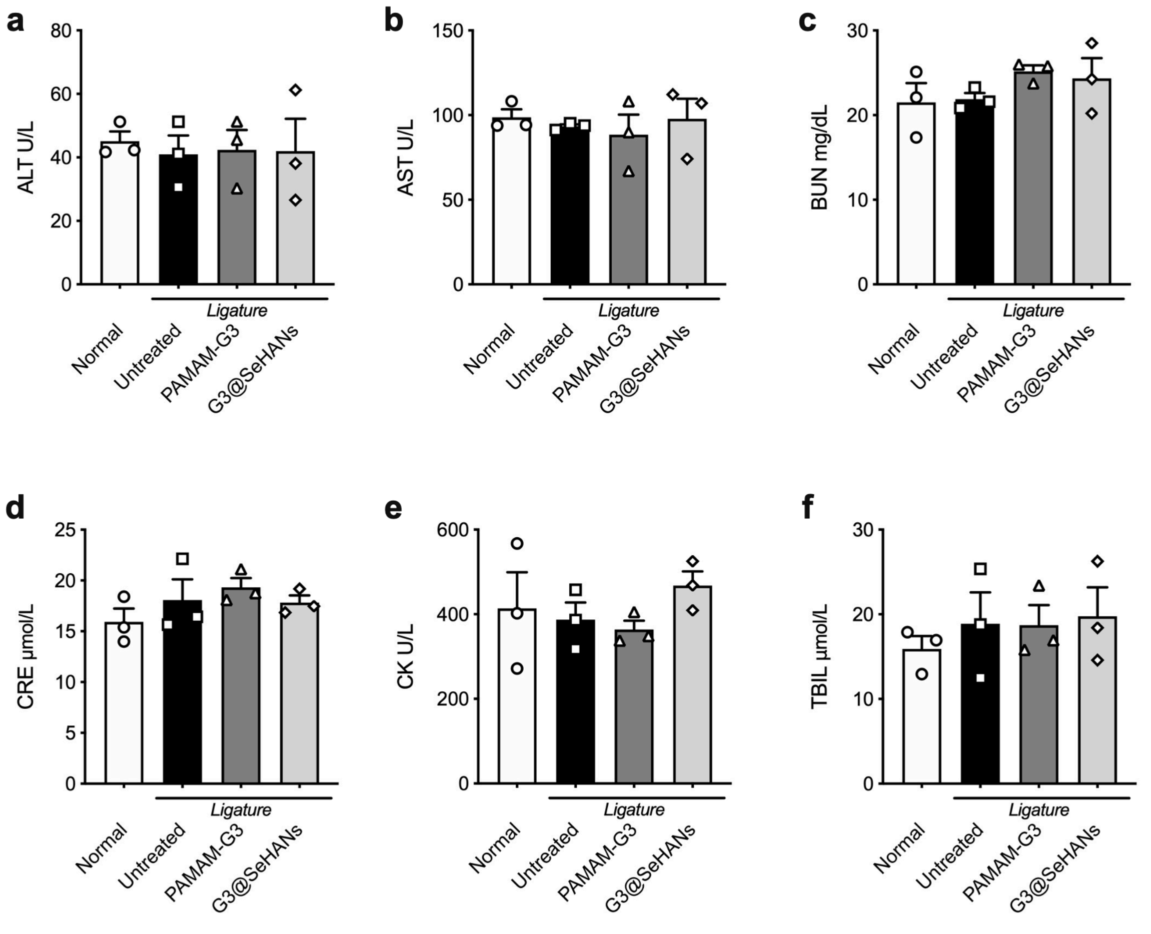


**Supplementary Fig. 26. Blood serum biochemistry parameters of mice.**

The blood serum biochemistry parameters (a) ALT, (b) AST, (c) BUN, (d) CRE, (e) CK, and (f) TBIL of the four groups were measured. One-way ANOVA with Tukey's multiple comparison tests (*n*=3 mice per group) were used, but no difference was found. Data are means ± SEM.

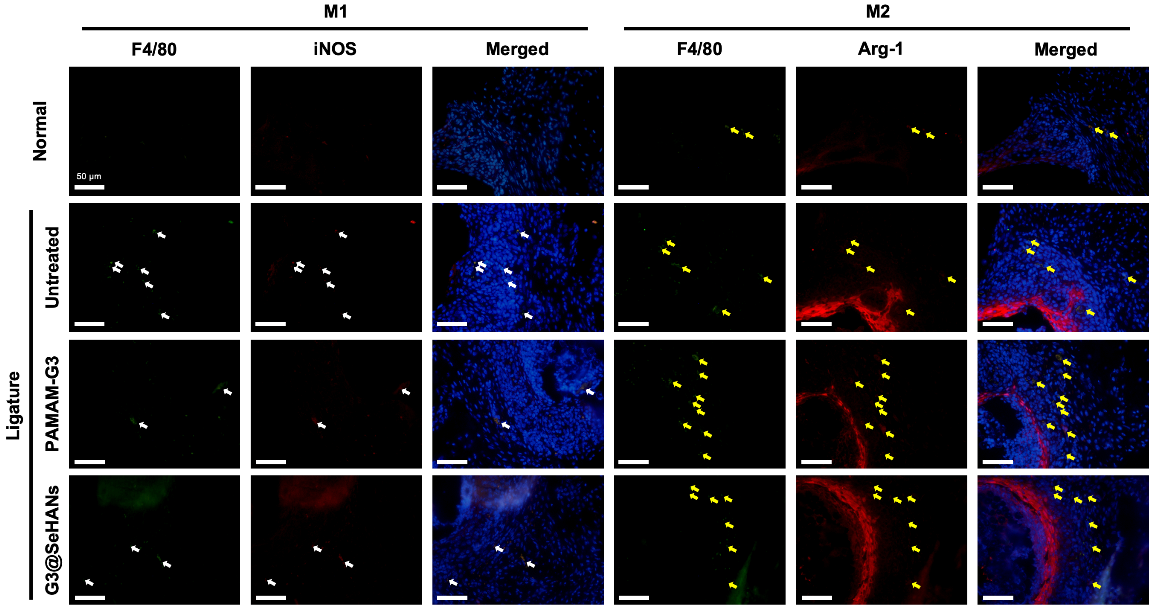


**Supplementary Fig. 27. IF staining showing polarization of macrophages in periodontal tissues on day 15.**

Macrophages were detected with F4/80 (green). M1-type macrophages were marked with iNOS (red in the M1 column), and M2-type macrophages were marked with arginase-1 (red in the M2 column). DAPI was used to stain the cell nucleus (blue). Barely any M1 macrophages were found in the normal group, but the number of M1 macrophages was higher in the untreated group than the cfDNA scavenger-treated groups. In contrast, the number of M2 macrophages increased in the cfDNA scavenger-treated groups. Scale bars, 50 μm. White arrows, $F4/80^{+}iNOS^{+}$ cells (M1); yellow arrows, $F4/80^{+}Arg\text{-}1^{+}$ cells (M2). Images were representative of three independent mice.

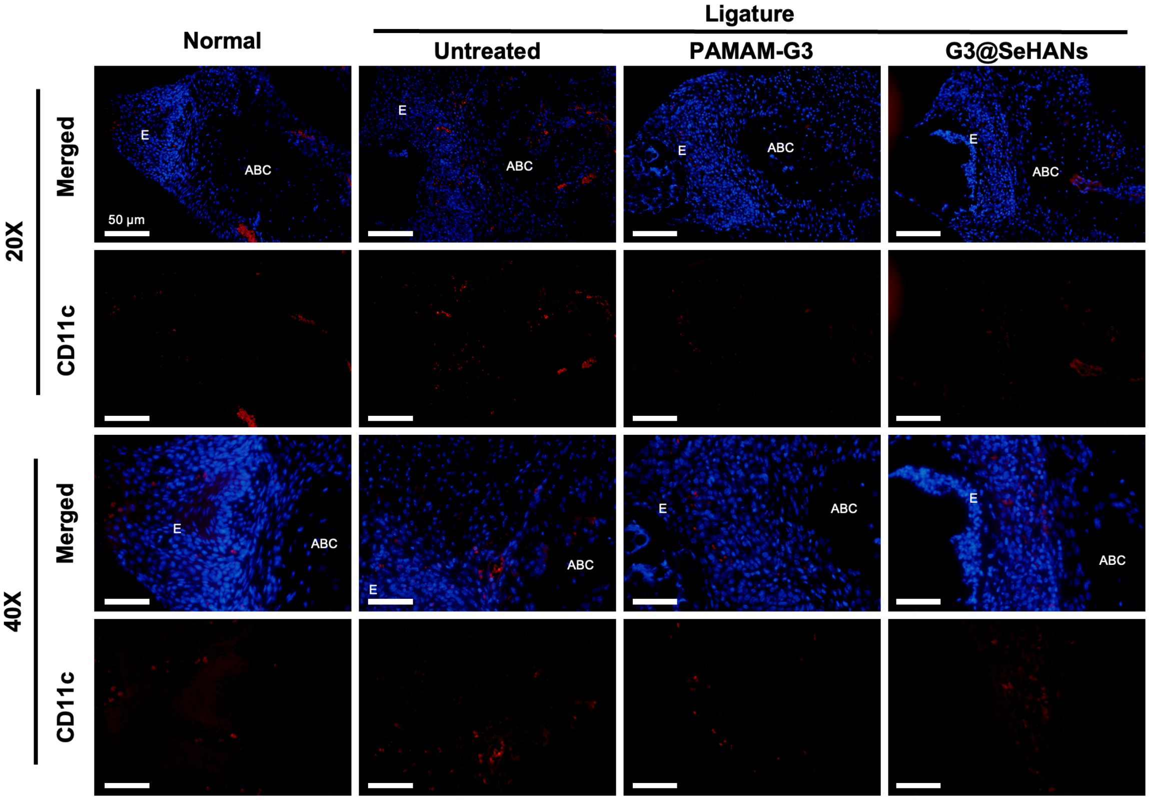


**Supplementary Fig. 28. IF staining of DCs (CD11c) in periodontal tissues on day 15.**

CD11c IF staining (red) showed the number of DCs in four groups. E, epithelium; ABC, alveolar bone crest. Scale bars, 50 μm. Images were representative of three independent mice.

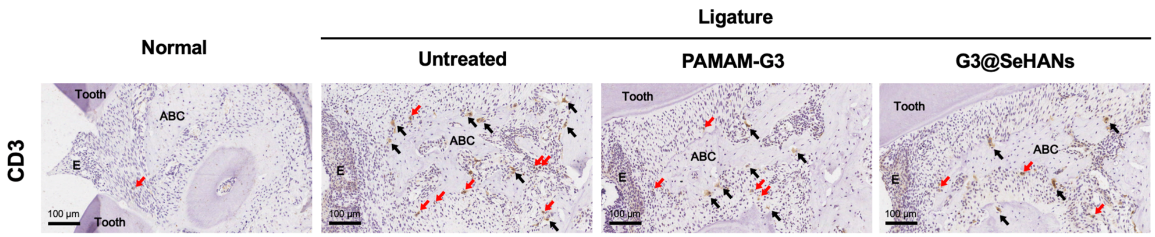


**Supplementary Fig. 29. IHC staining of T cells (CD3) in periodontal tissues on day 15.**

IHC staining of T cells (CD3) in periodontal tissues on day 15 after G3@SeHANs administration. Scale bars, 100 µm. CD3 IHC staining that the increased number of T cells in the untreated group could be controlled by scavenger treatment. Black arrows, osteoclasts; Red arrows, T cells; E, epithelium; ABC, alveolar bone crest. Images were representative of three independent mice.

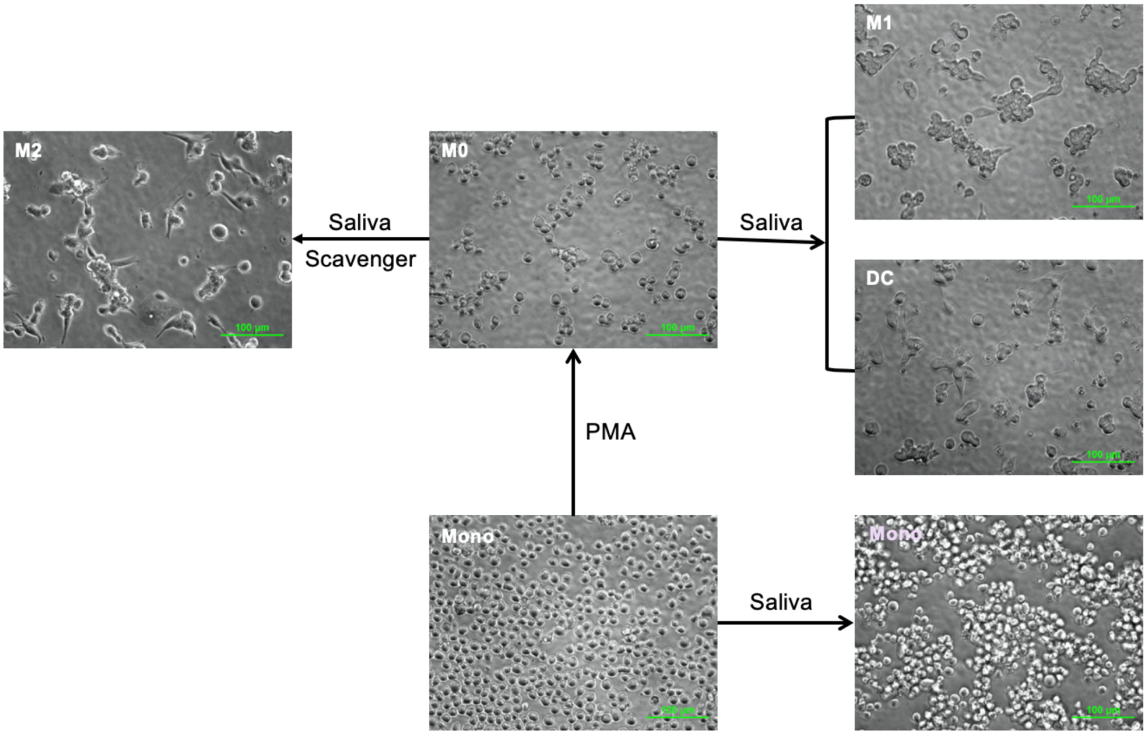


**Supplementary Fig. 30. Morphology of THP-1 cells treated with PMA, periodontitis saliva, and scavengers.**

Mono, monocytes; M0, M0-phenotype macrophage; M1, M1-phenotype macrophage; M2, M2-phenotype macrophage; DC, dendritic cell. Scale bar, 100 µm. Representative images were from 3 independent experiments.

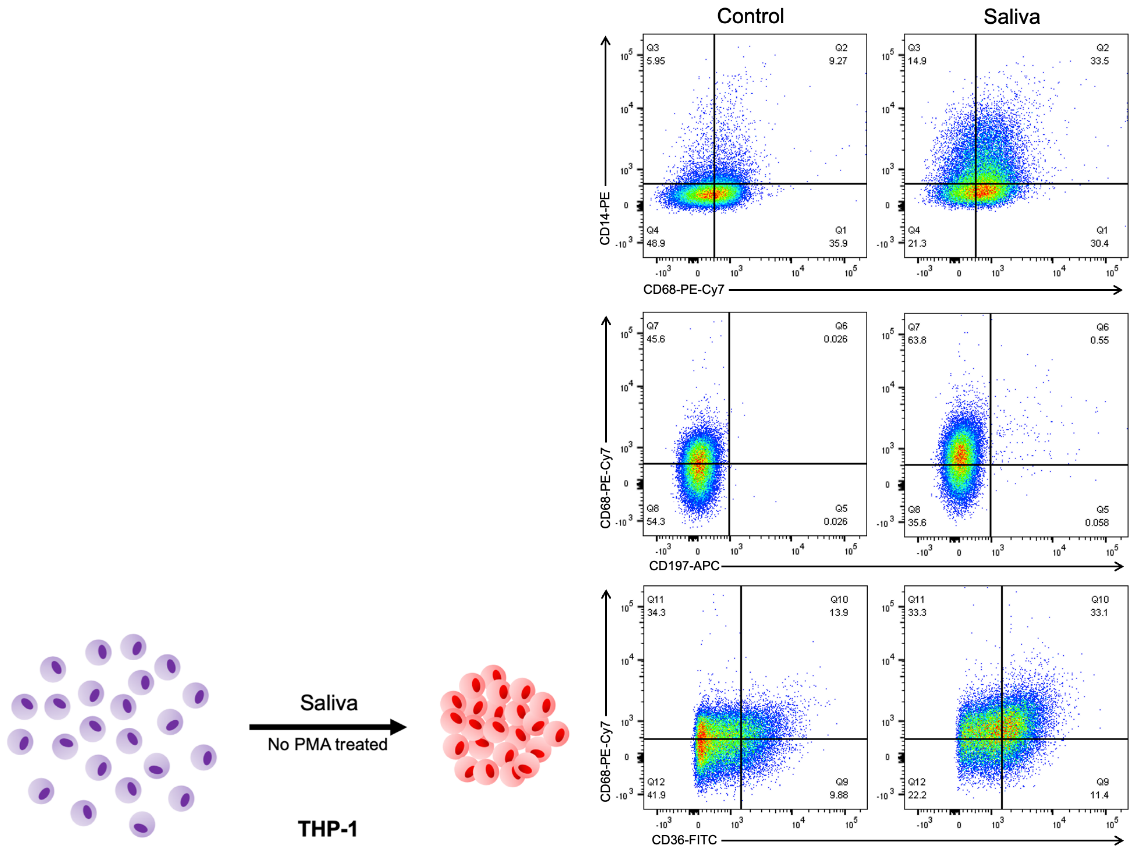


**Supplementary Fig. 31. THP-1 cells treated with periodontitis saliva without PMA.**

THP-1 cells were agglomerated after incubation with periodontitis saliva. Flow cytometry of cell markers (CD14, CD68, CD197, and CD36) was performed.

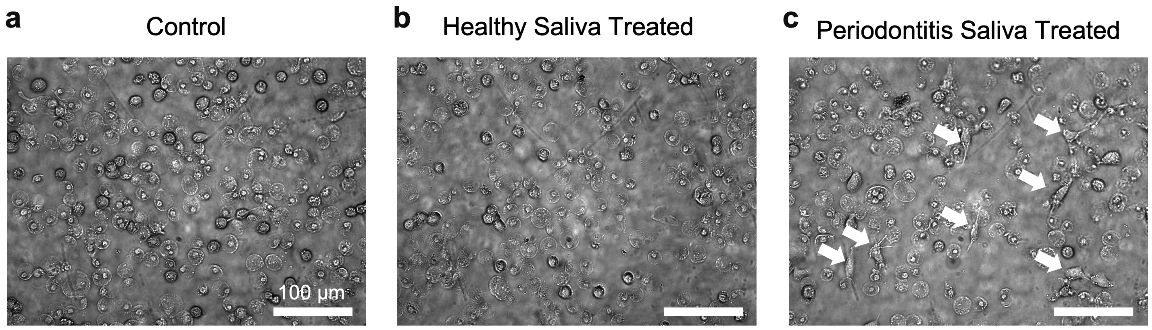


**Supplementary Fig. 32. Morphological changes of THP-1 cells incubated with healthy saliva and periodontitis saliva.**

Morphology of THP-1 cells incubated with (a) only PMA, (b) PMA and healthy saliva, and (c) PMA and periodontitis saliva. Arrows show significant morphological changes in cells. Scale bar, 100 μm. Representative images were from 3 independent experiments.

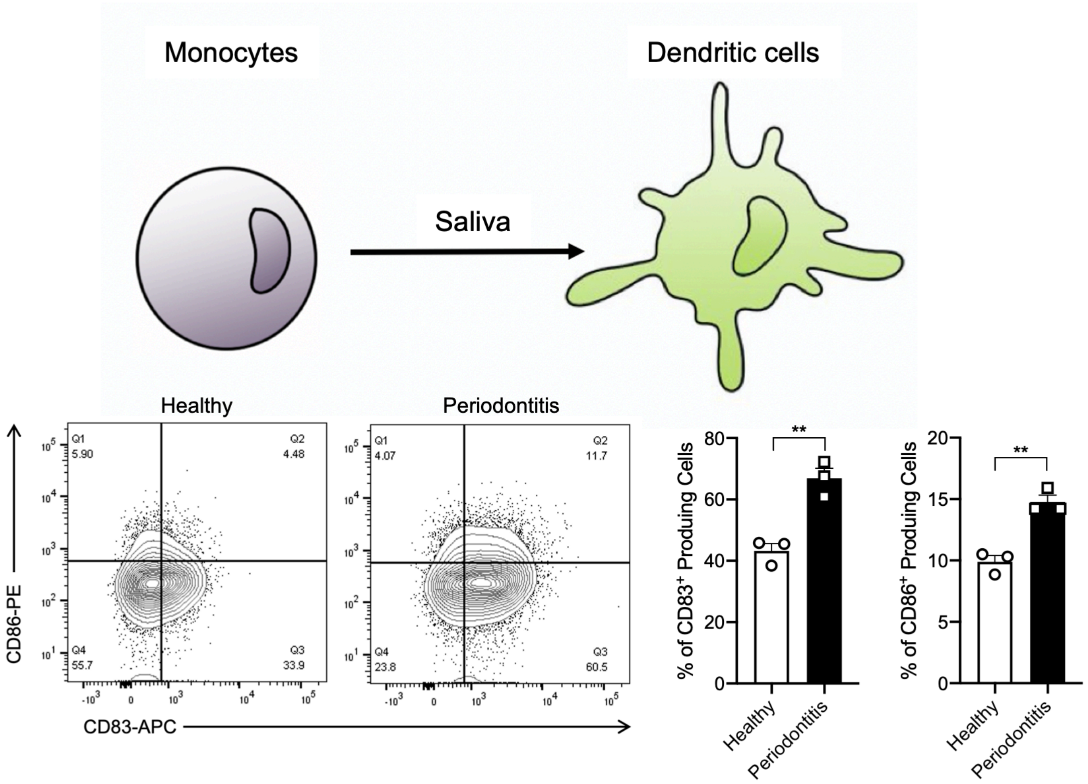


**Supplementary Fig. 33. DC differentiation of THP-1 cells treated with periodontitis saliva and PMA.**

THP-1 cells showed the potential to differentiate into DCs after incubation with periodontitis saliva. Flow cytometry of cell markers (CD86 and CD83) was performed. Data are means ± SEM (n=3 samples per group; **P*<0.05, ***P*<0.01 by two-tailed Student's *t* test) (CD83, *P*=0.0044; CD86, *P*=0.0031).

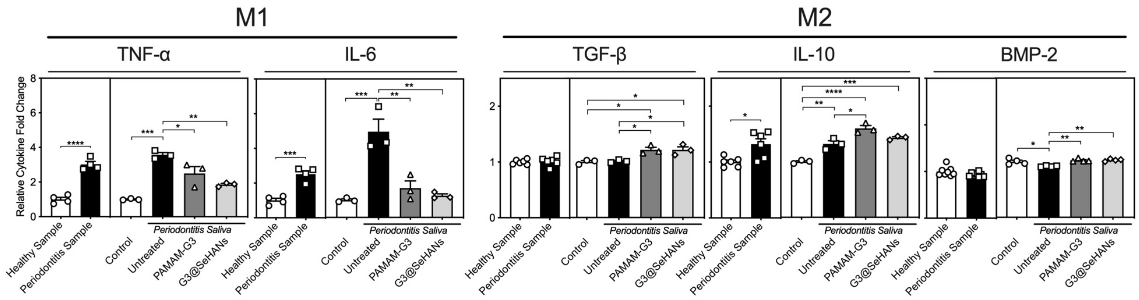


**Supplementary Fig. 34. Cytokine expression related to M1 and M2 macrophage phenotypes after incubation with periodontitis saliva and cfDNA scavengers.**

Activation of THP-1 cells by healthy human saliva or periodontitis patient saliva in the absence or presence of PAMAM-G3 (2 μg/mL) or G3@SeHANs (10 μg/mL). Supernatants were assayed for TNF-α, IL-6, TGF-β, IL-10, and BMP-2 by ELISA. Differences were assessed with a two-tailed Student's *t*-test and one-way ANOVA with Tukey's multiple comparison test. Data are means ± SEM ($n$=3 independent experiments). *$P$<0.05, **$P$<0.01, ***$P$<0.001, ****$P$<0.0001 (TNF-α, $P$<0.0001 between Healthy Sample and Periodontitis Sample, $P$=0.0001 between Control and Untreated, $P$=0.0299 between Untreated and PAMAM-G3, $P$=0.0020 between Untreated and G3@SeHANs; IL-6, $P$=0.0007 between Healthy Sample and Periodontitis Sample, $P$=0.0008 between Control and Untreated, $P$=0.0027 between Untreated and PAMAM-G3, $P$=0.0012 between Untreated and G3@SeHANs; TGF-β, $P$=0.0170 between Control and PAMAM-G3, $P$=0.0170 between Control and G3@SeHANs, $P$=0.0222 between Untreated and PAMAM-G3, $P$=0.0222 between Untreated and G3@SeHANs; IL-10, $P$=0.0104 between Healthy Sample and Periodontitis Sample, $P$=0.0025 between Control and Untreated, $P$<0.0001 between Control and PAMAM-G3, $P$=0.0003 between Control and G3@SeHANs, $P$=0.0058 between Untreated and PAMAM-G3; BMP-2, $P$=0.0140 between Control and Untreated, $P$=0.0021 between Untreated and PAMAM-G3, $P$=0.0016 between Untreated and G3@SeHANs).

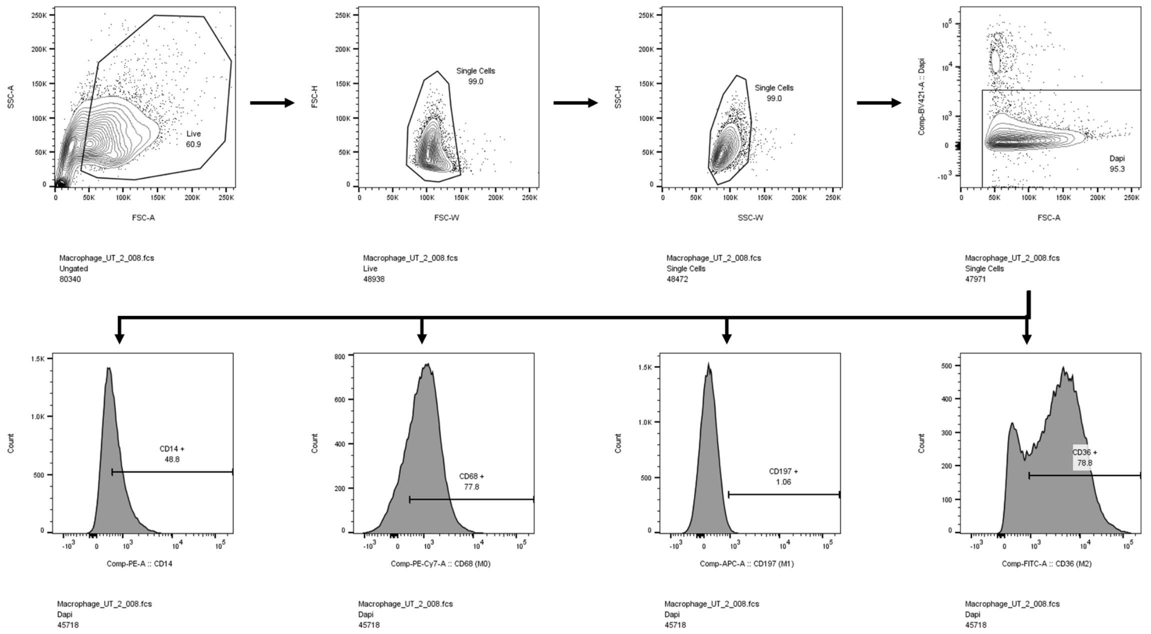


**Supplementary Fig. 35. Exemplification of the gating strategy for flow cytometry analysis.**

**Supplementary Tab. 1. Patient demographics**

| | Normal (N=10) | Gingivitis (Gin, N=10) | Periodontitis (CP, N=10) |
|---|---|---|---|
| Age (mean, range) | 29.7 (24-57) | 28.3 (20-35) | 43.3 (25-62) |
| Gender (M/F) | 3/7 | 4/6 | 3/7 |
| BMI* (mean ± SD) | 21.4±3.5 | 21.8±2.6 | 23.4±3.5 |
| Brushing ≥ 2 times/day (Y/N) | 10/0 | 8/2 | 9/1 |
| Bass (Y/N) | 10/0 | 8/2 | 3/7 |
| Mouthwash (Y/N) | 0/10 | 0/10 | 5/5 |
| Flossing (Y/N) | 8/2 | 5/5 | 4/6 |
| Smoking | 0 | 0 | 0 |
| Drinking | 0 | 0 | 0 |

*BMI, body mass index.

**Supplementary Tab. 2. ANOSIM analysis of three groups at the OTU level.**

| Comparison between groups | $R^2$ | *P* value |
|---|---|---|
| Untreated vs PAMAM-G3 | 0.1875 | 0.156 |
| Untreated vs G3@SeHANs | 0.1354 | 0.296 |
| PAMAM-G3 vs G3@SeHANs | 0.1146 | 0.336 |
| Untreated vs PAMAM-G3 vs G3@SeHANs | 0.1343 | 0.123 |

**Supplementary Tab. 3. Primers for qRT-PCR.**

| Primer | Forward | Reverse |
|---|---|---|
| *Gapdh* | AGGTTGTCTCCTGCGACTTCA | CCAGGAAATGAGCTTGACAAA |
| *Tlr9* | TTCTCAAGACGGTGGATCGC | GCAGAGGGTTGCTTCTCACG |
| *Myd88* | CATGGTGGTGGTTGTTTCTGAC | TGGAGACAGGCTGAGTGCAA |
| *Traf6* | TGTTCTTAGCTGCTGGGGTGT | GAAGGAGCTGGAGAGGTTCC |
| *Map3k7* | ACAACATTGTAAAATGGCACAGGAG | TTTTGCTGGTCCTTTTCATCCT |
| *Irf7* | GAACTTAGCCGGGAGCTTGG | TGGAGCCCAGCATTTTCTCTT |
| *Rela* | TTCCTGGCGAGAGAAGCAC | AAGCTATGGATACTGCGGTCT |
| *Tnf* | AGGGTCTGGGCCATAGAACT | CCACCACGCTCTTCTGTCTAC |
| *Il6* | CTCTGCAAGAGACTTCCATCCAGT | GAAGTAGGGAAGGCCGTGG |
