# Supplementary material for: Nanoparticulate cell-free DNA scavenger for treating inflammatory bone loss in periodontitis
Source: Nat Commun. 2022 Oct 7;13:5925. doi: 10.1038/s41467-022-33492-6 (PMC9546917; doi:10.1038/s41467-022-33492-6)
Supplement: Supplementary file 3 — Reporting Summary [file 41467_2022_33492_MOESM3_ESM.pdf]

nature portfolio

Corresponding author(s): Kam W. Leong

Last updated by author(s): Sep 15, 2022

Reporting Summary

Nature Portfolio wishes to improve the reproducibility of the work that we publish. This form provides structure for consistency and transparency in reporting. For further information on Nature Portfolio policies, see our Editorial Policies and the Editorial Policy Checklist.

Statistics

For all statistical analyses, confirm that the following items are present in the figure legend, table legend, main text, or Methods section.

n/a	Confirmed	
☐	☒	The exact sample size (*n*) for each experimental group/condition, given as a discrete number and unit of measurement
☐	☒	A statement on whether measurements were taken from distinct samples or whether the same sample was measured repeatedly
☐	☒	The statistical test(s) used AND whether they are one- or two-sided<br>*Only common tests should be described solely by name; describe more complex techniques in the Methods section.*
☒	☐	A description of all covariates tested
☒	☐	A description of any assumptions or corrections, such as tests of normality and adjustment for multiple comparisons
☐	☒	A full description of the statistical parameters including central tendency (e.g. means) or other basic estimates (e.g. regression coefficient) AND variation (e.g. standard deviation) or associated estimates of uncertainty (e.g. confidence intervals)
☐	☒	For null hypothesis testing, the test statistic (e.g. *F*, *t*, *r*) with confidence intervals, effect sizes, degrees of freedom and *P* value noted<br>*Give P values as exact values whenever suitable.*
☒	☐	For Bayesian analysis, information on the choice of priors and Markov chain Monte Carlo settings
☒	☐	For hierarchical and complex designs, identification of the appropriate level for tests and full reporting of outcomes
☒	☐	Estimates of effect sizes (e.g. Cohen's *d*, Pearson's *r*), indicating how they were calculated

Our web collection on statistics for biologists contains articles on many of the points above.

Software and code

Policy information about availability of computer code

Data collection	Data were collected with various laboratory instruments and equipment. Such as: Laser confocal microscopy, LSRFortessa or LSR II for flow cytometry, Aperio ScanScope slide scanner, Upright Automated Fluorescence Microscope, micro-CT scanning, Zetasizer, scanning electron microscopy, field emission transmission electron microscopy, Fourier-transform infrared spectroscopy, X-ray photoelectronic spectroscopy, etc.
Data analysis	GraphPad Prism 8, ggplot2 (3.3.5) and pheatmap (1.0.12) package on R (version 4.1.1), FlowJo software v10.8.1, Image-Pro Plus v6.0.0, Photoshop CC, Mimics Research v19.0.0, Mothur (v.1.43.0), QIIME 2.0, USEARCH (v10)

For manuscripts utilizing custom algorithms or software that are central to the research but not yet described in published literature, software must be made available to editors and reviewers. We strongly encourage code deposition in a community repository (e.g. GitHub). See the Nature Portfolio guidelines for submitting code & software for further information.

Data

Policy information about availability of data

All manuscripts must include a data availability statement. This statement should provide the following information, where applicable:

- Accession codes, unique identifiers, or web links for publicly available datasets
- A description of any restrictions on data availability
- For clinical datasets or third party data, please ensure that the statement adheres to our policy

All the data generated in this study are provided in the Supplementary Information/Source Data file. All 16S rRNA genes sequences and genomes generated in the present study are available via Sequence Read Archive (SRA) using the individual accession numbers (PRJNA779377 [https://www.ncbi.nlm.nih.gov/bioproject/PRJNA779377]).

Field-specific reporting

Please select the one below that is the best fit for your research. If you are not sure, read the appropriate sections before making your selection.

- [x] Life sciences
- [ ] Behavioural & social sciences
- [ ] Ecological, evolutionary & environmental sciences

For a reference copy of the document with all sections, see nature.com/documents/nr-reporting-summary-flat.pdf

Life sciences study design

All studies must disclose on these points even when the disclosure is negative.

Sample size	Sample sizes were chosen based on previously published literature and protocols in the field with a similar setup that showed statistical significance (Human: 10.1126/sciadv.aay7148; Mouse: 10.1111/jcpe.13060). For each animal experiment, we have adopted at least n=3 biological replicates to calculate the statistical value of each analysis and the exact sample sizes and statistical data for each experiment are reported in the figure legends.
Data exclusions	No data were excluded from the analyses.
Replication	All experiments were repeated at least three times with reproducibility. All attempts at replication were successful and the exact numbers are indicated in the figure legends or table for all experiments.
Randomization	Human participants were allocated into different groups according to the periodontal clinical diagnosis. To control potential covariates, potential participants with systematic disease, history of smoking or betel nut chewing were excluded; a consistent time and protocol of sample collection were used; and baseline demographic information was counted and reported for each group of participants. Mice were assorted randomly to cages when received from the vendor.
Blinding	The investigators were blinded to group allocation during data collection and analysis.

Reporting for specific materials, systems and methods

We require information from authors about some types of materials, experimental systems and methods used in many studies. Here, indicate whether each material, system or method listed is relevant to your study. If you are not sure if a list item applies to your research, read the appropriate section before selecting a response.

Materials & experimental systems

n/a	Involved in the study	
[ ]	[x]	Antibodies
[ ]	[x]	Eukaryotic cell lines
[x]	[ ]	Palaeontology and archaeology
[ ]	[x]	Animals and other organisms
[ ]	[x]	Human research participants
[x]	[ ]	Clinical data
[x]	[ ]	Dual use research of concern

Methods

n/a	Involved in the study	
[x]	[ ]	ChIP-seq
[ ]	[x]	Flow cytometry
[x]	[ ]	MRI-based neuroimaging

Antibodies

Antibodies used

Antibodies used in IF/IHC:
Anti-CD11c (N418) Armenian hamster monoclonal antibody (1:100; eBioscience, cat. #14-0114-82)
Anti-F4/80 (BM8) rat monoclonal antibody (1:50; eBioscience, cat. #14-4801-82)
Anti-iNOS (D6B6S) rabbit monoclonal antibody (1:400; Cell Signaling Technology, cat. #13120)
Anti-arginase-1 (D4E3M) XP rabbit monoclonal antibody (1:50; Cell Signaling Technology, cat. #93668)
Anti-CD3 rabbit polyclonal antibody (1:100; Abcam, cat. #ab5690)
Anti-TLR9 rabbit polyclonal antibody (1:500; Sigma-Aldrich, cat. #SAB3500313)
Alexa Fluor 647-conjugated goat anti-Armenian hamster IgG antibody (1:100; Abcam, cat. #ab173004)
Dylight 488-conjugated AffiniPure goat anti-rat IgG (H+L) (1:100; EARTHOX, cat. #E032240)
Alexa Fluor 594-conjugated goat anti-rabbit IgG (H+L) (1:100; ZSGB-BIO, cat. #ZF-0516)

Antibodies used in pull-down:
ChIP-grade anti-histone H3 rabbit polyclonal antibody (1:200; Abcam, cat. #ab1791)

Antibodies used in flow cytometry (1:20) :
APC anti-human CD83 monoclonal antibody (HB15e), (eBioscience, cat. #17-0839-4)

PE anti-human CD86 monoclonal antibody (IT2.2) (eBioscience, cat. #12-0869-41)
PE-Cy7 anti-human CD209 monoclonal antibody (eB-h209) (eBioscience, cat. #25-2099-41)
PE anti-human CD14 monoclonal antibody (61D3) (eBioscience, cat. #12-0149-41)
PE-Cy7 anti-human CD68 monoclonal antibody (Y1/82A) (eBioscience, cat. #25-0689-41)
APC anti-human CD197 monoclonal antibody (3D12) (eBioscience, cat. #17-1979-41)
FITC anti-human CD14 monoclonal antibody (HCD14) (BioLegend, cat. #325603).
FITC anti-human CD36 monoclonal antibody (CB38) (BD Biosciences, cat. #555454).

Validation

All antibodies used in experiments are commercially available and have been validated by the manufacturer. All validation statements can be found on the respective antibody website. In house validations were performed before using antibodies with new log number. Clones of antibodies used in flow cytometry were chosen based on published literature.

Eukaryotic cell lines

Policy information about cell lines

Cell line source(s)	Human gingival keratinocytes (HGKs), human gingival fibroblasts (HGFs), THP-1, and RAW 264.7 were purchased from ATCC. hTLR3-, 4-, 8-, and 9-overexpressing HEK-Blue cells were purchased from InvivoGen.
Authentication	Authentication was performed by analysis of morphology.
Mycoplasma contamination	All cell lines tested negative for mycoplasma contamination.
Commonly misidentified lines (See ICLAC register)	No commonly misidentified lines were used in this study.

Animals and other organisms

Policy information about studies involving animals; ARRIVE guidelines recommended for reporting animal research

Laboratory animals	Male BALB/C mice (7-8 weeks old) were purchased from Dossy Experimental Animals Co., Ltd. (Chengdu, Sichuan, China) and maintained at the Experimental Animal Center of West China Second University Hospital, Sichuan University (temperature: 18-29 °C, humidity: 40-70%, dark/light cycle: natural circadian rhythms).
Wild animals	This study did not involve use of wild animals.
Field-collected samples	This study did not involve use of field-collected samples.
Ethics oversight	The animal study was approved (WCHSIRB-D-2020-498) by the Ethics Committee of West China School of Stomatology (Chengdu, China).

Note that full information on the approval of the study protocol must also be provided in the manuscript.

Human research participants

Policy information about studies involving human research participants

Population characteristics	Specific population characteristics were shown in Tab. S1.
Recruitment	Health volunteers were recruited from the community or healthy companions of patients, and participants with gingivitis and periodontitis were recruited from the consecutive patients attending Department of Periodontics, West China Hospital of Stomatology, Sichuan University. As participant compensation, participants who completed sample collection received a complete oral examination and, if needed, periodontal treatment for free.<br>To control selection bias, gingivitis and periodontitis was diagnosed strictly according to the 2018 new classification and definition of periodontitis, as well as the definition of periodontal clinical healthy control. In addition, after periodontal clinical examination and confirmation of enrollment, consistent time (09:00 AM) was scheduled for sample collection, and participants were informed to avoid eating, drinking and oral hygiene cleaning on the same day before sample collection.
Ethics oversight	Patient sample collection was performed with the approval (WCHSIRB-D-2020-461) of the Ethics Committee of West China Hospital of Stomatology, Sichuan University. All participants signed an informed consent form prior to sample collection and were informed of the potential benefits and risks of participating.

Note that full information on the approval of the study protocol must also be provided in the manuscript.

Flow Cytometry

Plots

Confirm that:

☒ The axis labels state the marker and fluorochrome used (e.g. CD4-FITC).

☒ The axis scales are clearly visible. Include numbers along axes only for bottom left plot of group (a 'group' is an analysis of identical markers).

☒ All plots are contour plots with outliers or pseudocolor plots.

☒ A numerical value for number of cells or percentage (with statistics) is provided.

Methodology

Sample preparation	THP-1 cells were treated with the method described in the in vitro anti-inflammatory assay section and were collected for flow cytometry.
Instrument	LSRFortessa or LSR II (BD Biosciences)
Software	FlowJo software (BD Biosciences)
Cell population abundance	Roughly 1*10E6 THP-1 cells as one test of flow cytometry.
Gating strategy	Cells were gated as following: FSC-A/SSC-A, FSC-W/FSC-H, SSC-W/SSC-H, Dapi negative, and markers for dendritic cells or monocytes or macrophages as described in the Materials and Methods section.

☒ Tick this box to confirm that a figure exemplifying the gating strategy is provided in the Supplementary Information.
